# Association Between Maternal Infections in Pregnancy and the Risk of Inflammatory Bowel Disease in the Offspring: Findings From Two Scandinavian Birth Cohorts

**DOI:** 10.1093/ibd/izae209

**Published:** 2024-09-09

**Authors:** Annie Guo, Johnny Ludvigsson, Tereza Lerchova, Henrik Imberg, Ketil Størdal, Karl Mårild

**Affiliations:** Department of Pediatrics, Institute of Clinical Sciences, Sahlgrenska Academy, University of Gothenburg, Gothenburg, Sweden; Crown Princess Victoria Children’s Hospital, Region Östergötland, Linköping, Sweden; Division of Pediatrics, Department of Biomedical and Clinical Sciences, Linköping University, Linköping, Sweden; Department of Pediatrics, Institute of Clinical Sciences, Sahlgrenska Academy, University of Gothenburg, Gothenburg, Sweden; Statistiska Konsultgruppen, Gothenburg, Sweden; Department of Molecular and Clinical Medicine, Institute of Medicine, Sahlgrenska Academy, University of Gothenburg, Gothenburg, Sweden; Children’s Center, Oslo University Hospital, Oslo, Norway; Department of Pediatric Research, Faculty of Medicine, University of Oslo, Oslo, Norway; Department of Pediatrics, Institute of Clinical Sciences, Sahlgrenska Academy, University of Gothenburg, Gothenburg, Sweden; Department of Pediatrics, Queen Silvia Children’s Hospital, Gothenburg, Sweden

**Keywords:** inflammatory bowel disease, ABIS, MoBa

## Abstract

**Background:**

The association of infections and antibiotic use in pregnancy and the risk of inflammatory bowel disease (IBD) development in the offspring have been scarcely investigated. We examined infection and antibiotic use in pregnancy and the risk of IBD in offspring.

**Methods:**

We followed participants from the All Babies in Southeast Sweden (ABIS) and the Norwegian mother father and child cohort (MoBa) from birth (1997-2009) until 2020-2021. IBD diagnosis was classified as ≥2 records in national registers. Information on infections (any, gastrointestinal, and respiratory), their timing (early or late in pregnancy), and antibiotic use in pregnancy were collected from questionnaires. Cox proportional-hazard regression and meta-analytic methods were used to estimate pooled adjusted hazard ratios (aHRs) for IBD and its subtypes, adjusted for parental IBD, maternal smoking, and education. Sensitivity analyses accounted for exposure to antibiotics and infections 0-12 months of age.

**Results:**

We followed 117 493 children for 2 024 299 person-years (follow-up 22.3 years in ABIS and 16.4 years in MoBa), including 451 IBD cases. The aHRs for any infection and respiratory infections in pregnancy and offspring IBD were close to one (aHR = 0.99 [95% CI = 0.73-1.33] and aHR = 1.00 [95% CI = 0.81-1.23], respectively). However, any versus no infection in early pregnancy was associated with IBD development (aHR = 1.26 [95% CI = 1.02-1.55]), particularly Crohn’s disease (CD; aHR = 1.40 [95% CI = 1.01-1.93]). Any versus no gastrointestinal infection in late pregnancy was associated with offspring CD (aHR = 1.95 [95% CI = 1.34-2.84]). Antibiotic use in pregnancy was not associated with IBD in the child (aHR = 1.15 [95% CI = 0.93-1.44]).

**Conclusions:**

In this binational birth cohort study, the risk of offspring IBD varied by infection type and timing but not with maternal antibiotic use in pregnancy.

**Key Messages**

**What is already known?**

Early-life environmental factors, including exposure to infections and antibiotics, have been suggested to contribute to the rising incidence of inflammatory bowel disease (IBD).

**What is new here?**

Using prospectively collected binational cohort data, maternal infection in early pregnancy and gastrointestinal infection in late pregnancy were linked to an increased risk of IBD in offspring, specifically Crohn’s disease, regardless of the mother’s and child’s exposure to antibiotics.

**How can this help patient care?**

This study highlights the importance of maternal infections in pregnancy for the offspring’s risk of IBD, which may be useful for physicians in the future prevention of IBD.

## Introduction

Inflammatory bowel disease (IBD) comprises 2 main subtypes: Crohn’s disease (CD) and ulcerative colitis (UC). The occurrence of the disease is a result of the interplay between genetic and environmental factors, which may converge through their effects on the gut microbiome.^1,2^ Previous studies have underscored the significance of early-life exposures, particularly during pregnancy, in relation to the development of IBD.^3^

Maternal infections and antibiotic use during pregnancy have the potential to significantly impact the gut microbiome of offspring.^4^ This can have implications for the development of the child’s immune system and may also influence their long-term susceptibility to IBD.^5^ However, there is a lack of sufficient and consistent data on maternal exposure to infections and antibiotics in pregnancy and the subsequent risk of IBD in offspring (Supplementary Table S1).^5-10^ No association has been observed between maternal infections in pregnancy and offspring risk of IBD,^5,9^ and the findings for antibiotic exposure are inconsistent.^5-8,10^ However, most studies have been restricted to retrospectively collected data.

Although infections and antibiotic use are highly related exposures, existing research has not examined them jointly concerning IBD risk in offspring.^6^ Using prospectively collected data from 2 Scandinavian birth cohorts, we examined the association of maternal infections and antibiotic use in pregnancy with IBD risk in offspring.

## Materials and Methods

### Study Population

**Figure 1.**
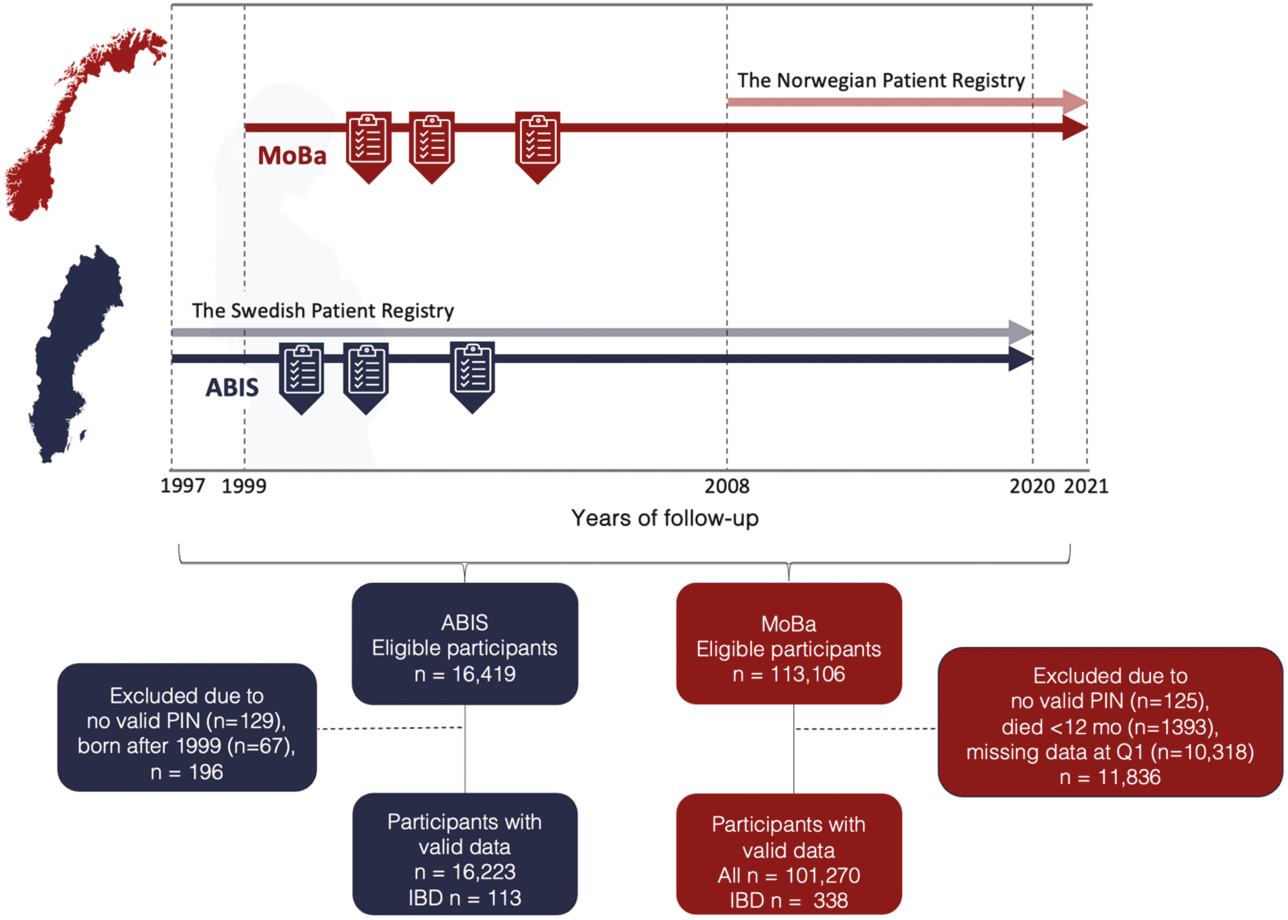
Flowchart of study participants included from the ABIS and MoBa cohorts. Because the ABIS target population was restricted to births in 1997-1999, we excluded children born after 1999. The Norwegian Patient Registry started in 1999 but only allowed linkage to the PIN from 2008. ABIS is a regional cohort study in Southeast Sweden; MoBa is a nationwide cohort in Norway. ABIS, All Babies in Southeast Sweden; IBD, inflammatory bowel disease; MoBa, The Norwegian Mother, Father, and Child Cohort study; PIN, personal identification number; Q1, baseline questionnaire administered at gestational week 15.

The All Babies in Southeast Sweden (ABIS) and the Norwegian Mother, Father, and Child Cohort Study (MoBa) follow children from birth to adulthood. Between October 1997 and October 1999, ABIS invited all 21 700 mothers and their newborn children from Southeast Sweden, of whom 17 055 consented to participate (participation rate 79%).^11^ MoBa, which recruited women in early pregnancy between July 1999 and December 2008,^12^ includes 114 000 children (participation rate 41%). This study was restricted to mothers and children with a valid personal identification number and data on baseline characteristics and exposures retrieved from questionnaires collected at birth (ABIS) and at gestational week (GW) 15 (MoBa; Figure 1). Our study used data from questionnaires collected during pregnancy and early childhood, in addition to data retrieved from national health registries (Supplementary Table S2).^13,14^

### Exposures

#### Maternal infections

At the child’s birth, the mothers in ABIS reported symptoms of gastrointestinal infection (GI) and any other infections experienced during pregnancy (yes/no). In a follow-up question, the mothers provided information regarding the specific month of pregnancy during which they were exposed to infection (ranging from 1 to 9 months). The mothers in MoBa reported infections in pregnancy through questionnaires distributed at GW 15, 30, and when the child reached 6 months of age (covering the period between GW 30 and birth). These self-reported data allowed us to capture any infection type, including mild infections that did not require medical attention. As shown in Supplementary Table S3, infections were categorized into any versus no respiratory infection (RI, specified as “common cold,” “throat infection,” “influenza,” “tonsillitis,” “ear infection,” “pneumonia,” or “bronchitis”), any versus no GI (“diarrhea, fever, and vomiting,” “gastric flu or diarrhea”), or any versus no overall infection, defined as any specified or unspecified infectious disease (including RI and GI) in pregnancy. The questionnaires were not designed to capture the number of infections in pregnancy.

#### Timing of maternal infections

Given that the mothers in ABIS were separately asked about the nature and timing of infection by month of pregnancy (Supplementary Table S3), we restricted our analysis to children whose mothers reported valid data on infection type and time point of infection. All participants in MoBa had complete data on the nature and timing of infections in pregnancy and were included in the analytical process. Based on previous studies,^15-17^ and the wording of the questionnaires in ABIS and MoBa, infections were classified as early pregnancy infections if reported within the first 16 weeks of pregnancy and as late pregnancy infections thereafter (ie, ≥17 pregnancy weeks). For example, early pregnancy infections were defined as reporting to have any infectious disease ≤4 months in the pregnancy in the 1-year questionnaire in ABIS and ≤16 pregnancy weeks in the questionnaires distributed at GW 15 and 30 in MoBa. A more detailed description is presented in Supplementary Table S3.

#### Maternal antibiotics

The mothers were instructed to complete questionnaires regarding their use of antibiotics (ABIS) in pregnancy and provide the names of any medications taken (MoBa; Supplementary Table S3). Reported medications in MoBa were categorized based on the time of usage in pregnancy and antibiotic classes using the anatomic therapeutic chemical pharmaceutical classification system.^18^ A similar definition was not possible in ABIS. Consequently, pooled analyses only permitted the investigation of any versus no antibiotics in pregnancy.

### Outcome

The diagnosis of IBD was determined by the presence of at least 2 International Statistical Classification of Diseases and Health Related Problems, 10th revision (ICD)-10 codes for IBD in the Swedish National Patient Register^19^ (ABIS) and the Norwegian Patient Registry (Supplementary Table S4).^14^ Upon reviewing medical records, this register-based diagnostic algorithm for IBD has shown a positive predictive value of 93%.^20,21^ Data on IBD diagnoses were captured until December 31, 2020, in ABIS and December 31, 2021, in MoBa. We used subtype-specific ICD codes for CD and UC. Participants with a mix of ICD codes for CD and UC over the past 5 years of follow-up were categorized as having IBD-unclassified (IBD-U). This category was not examined as an independent outcome but included within our broader definition of any IBD.

### Other Data

Drawing upon relevant literature,^3,6^ we have gathered information on factors that may influence the relationship between maternal infections, maternal antibiotic use, and the risk of offspring developing IBD (Supplementary Figure S1). These factors include the child’s sex (female/male), parental history of IBD (at least one parent with IBD), parental origin (the mother’s native language [MoBa]/the parent’s country of birth in [ABIS]), maternal smoking in pregnancy (yes/no), and maternal education level (education at the time of birth). Except in MoBa, where information on the child’s sex and parental history of IBD was retrieved from national registers, information on the other factors was collected through self-reported questionnaires administered from the pregnancy and up to the child’s age of 6 months (Supplementary Table S2).^13,14^ We performed additional adjustments for the duration of full breastfeeding (exclusive breastfeeding), the child’s own infection frequency and antibiotic consumption in the first year of life as well as delivery mode (vaginal/cesarean section). Information on the duration of breastfeeding was retrieved from self-reported questionnaires captured at the child’s age of 12 months in ABIS and 6 and 18 months in MoBa. Data on the child’s exposure to infections and antibiotics was collected at 12 months (ABIS) and 18 months of age (MoBa; Supplementary Figure S2). The delivery mode was collected from the Medical Birth Registry of Norway in MoBa and from the birth questionnaire in ABIS. Variables were categorized as shown in Table 1.

**Table 1.** Study characteristics of participants in the ABIS and MoBa cohorts.

	ABIS		MoBa	
	All *n* = 16 223	IBD events *n* = 113	All *n* = 101 270	IBD events *n* = 338
Child’s sex^a^				
Female	7821 (48.2)	52 (46.0)	49 400 (48.8)	146 (43.2)
Male	8402 (51.8)	61 (54.0)	51 870 (51.2)	192 (56.8)
Follow-up years^b^				
Mean (SD)	22.2 (1.0)	16.9 (3.7)	16.4 (2.2)	12.8 (3.8)
Median (IQR)	22.3 (21.8;22.8)	17.9 (15.0;19.6)	16.2 (14.7, 18.1)	13.2 (10.7, 15.5)
Parental origin^a^				
Swedish/Norwegian	14 142 (87.2)	97 (85.8)	93 082 (91.9)	306 (90.5)
* Missing*	381 (2.3)	1 (0.9)	2530 (2.6)	15 (4.4)
Maternal education level^a^				
−11 years	1358 (8.4)	12 (10.6)	8029 (7.9)	37 (10.9)
12 years	8813 (54.3)	64 (56.6)	29 735 (29.4)	99 (29.3)
≥13 years	5669 (34.9)	36 (31.9)	62 997 (62.2)	199 (58.9)
* Missing*	383 (2.4)	1 (0.9)	509 (0.5)	3 (0.9)
Parental IBD^a^^,^^c^	195 (1.2)	5 (4.4)	2325 (2.3)	9 (2.7)
* Missing*	0 (0)	(0)	0 (0)	(0)
Maternal comorbidities, yes^a^^,^^d^	568 (3.5)	4 (3.5)	4072 (4.0)	13 (3.8)
* Missing*	0 (0)	0 (0)	0 (0)	0 (0)
Maternal smoking^a^				
Yes	1760 (10.8)	13 (11.6)	9597 (9.5)	47 (13.9)
* Missing*	380 (2.3)	1 (0.9)	1587 (1.6)	7 (2.1)
Any maternal infections in pregnancy^a^^,^^e^				
Any time in pregnancy	8165 (50.3)	52 (46.0)	69 606 (68.7)	236 (69.8)
Early pregnancy	3984 (24.6)	29 (25.7)	49 504 (48.9)	178 (52.7)
Late pregnancy	5603 (34.5)	34 (30.1)	47 416 (46.8)	165 (48.8)
* Missing*	476 (2.9)	1 (0.9)	0 (0.0)	0 (0.0)
Maternal respiratory infection^a^^,^^e^				
Any time in pregnancy	1508 (9.3)	12 (10.6)	57 952 (57.2)	188 (55.6)
Early pregnancy	678 (4.2)	7 (6.2)	37 663 (37.2)	127 (37.6)
Late pregnancy	1019 (6.3)	7 (6.2)	38 937 (38.4)	125 (37.0)
Maternal gastrointestinal infection^a^^,^^e^				
Any time in pregnancy	5273 (32.5)	28 (24.8)	22 030 (21.8)	87 (25.7)
Early pregnancy	2395 (14.8)	15 (13.3)	12 070 (11.9)	45 (13.3)
Late pregnancy	3284 (20.2)	17 (15.0)	13 640 (13.5)	58 (17.2)
*Missing*	872 (5.4)	3 (2.7)	0 (0.0)	0 (0.0)
Maternal antibiotic use^a^^,^^e^				
Yes	2996 (18.5)	24 (21.2)	24 028 (23.7)	88 (26.0)
*Missing*	1434 (8.8)	8 (6.2)	0 (0.0)	0 (0.0)
Child’s infection 0-12 months^a^^,^^f^				
Yes, any infection	9569 (59.0)	72 (63.7)	82 208 (81.2)	278 (82.2)
*Missing*	6402 (39.5)	41 (36.3)	11 572 (11.4)	35 (10.4)
Child’s antibiotic use 0-12 months^a^^,^^f^				
Yes, any antibiotic use	3785 (23.3)	30 (26.5)	9390 (9.3)	44 (13.0)
*Missing*	5569 (34.3)	39 (34.5)	11 620 (11.5)	34 (10.1)
Delivery mode				
Vaginal delivery	13 040 (80.4)	91 (80.5)	86 028 (85.0)	275 (81.4)
Cesarean section	1866 (11.5)	15 (13.3)	15 242 (15.0)	63 (18.6)
Missing	1317 (8.1)	7 (6.2)	0 (0.0)	0 (0.0)

Data are presented as numbers and percentages if otherwise not specified. Analyses of the timing of infections are based on a subsample with valid data on exposure to infections and timepoint in pregnancy (any infection/respiratory infection, *n* = 109 145; gastrointestinal infections, *n* = 106 363).

^a^Data from questionnaires (Supplementary Table S2). Defined as mother’s native language (MoBa)/parent’s country of birth (ABIS).

^b^Data from the Swedish National Patient Register (ABIS).

^c^Data from the Medical Birth Registry of Norway and The Norwegian Patient Registry (MoBa).

^d^Type 1 diabetes (insulin-treated diabetes before or in pregnancy (MoBa) or type 1 diabetes/insulin-treated diabetes [ABIS]), autoimmune thyroid disease, and rheumatoid arthritis.

^e^Reported at the child’s birth (ABIS) or gestational weeks 15 and 30 and 6 months after the child’s birth (MoBa). Early pregnancy was defined as the first ≤4 months of pregnancy (ABIS) and gestational week ≤16 (MoBa). Late pregnancy was defined as the last ≥5 months of pregnancy (ABIS) and gestational week ≥17 (MoBa).

^f^Reported at the child’s age of 12 months (ABIS) and 18 months (MoBa) and was categorized as exposed or unexposed.

^g^Data from questionnaires (ABIS) and from the Medical Birth Registry of Norway (MoBa; Supplementary Table S2).

IBD, inflammatory bowel disease; IQR, interquartile range; SD, standard deviation.

### Statistical Analyses

Cox regression was performed to calculate hazard ratios (HRs) and 95% confidence intervals (CIs) for IBD and its subtypes. Poisson regression was applied to estimate the incidence rates for IBD. While we analyzed IBD, including events diagnosed with CD, UC, and IBD-U, and performed sub-analyses for the outcome of CD and UC, we decided not to assess IBD-U as a separate outcome owing to the higher risk of misclassification. In analyses for the outcome CD, events of UC and IBD-U were ignored and vice versa for UC analyses. The follow-up period commenced at the child’s birth and concluded either upon the first (minimum of 2) recorded diagnosis of IBD or upon censoring by the end of the study follow-up (December 31, 2020, for ABIS and December 31, 2021, for MoBa). The proportional-hazard assumption was evaluated using the Schoenfeld residuals, and interactions-with-time analyses, and found valid for all analyses. Because some mothers had more than one child in the same cohort, cluster-corrected (robust) standard errors were used. All analyses were adjusted for the child’s sex, parental IBD, parental origin, maternal smoking in pregnancy, and education level.

For significant associations, we (1) performed mutual adjustments for infection and antibiotic exposure in pregnancy, (2) adjusted analyses for full breastfeeding duration, and (3) examined mediating effects through the child’s infection or antibiotic exposure in the first 12 months of life. In sensitivity analyses, we restricted our analyses to childhood-onset IBD (<18 years) and excluded events of very early onset IBD (VEO-IBD; <6 years). We also adjusted maternal infections and antibiotic use and IBD development in the offspring for delivery mode. Statistical analyses were performed using the R statistical software version 4.1.3 (R Core Team, Vienna, Austria) version 4.1.3, including the packages survival (3.4-0), survminer (0.4.9), meta (1.2-0), and metafor (3.8-1), and IBM SPSS Statistics (IBM Corp. Armonk, NY) version 29.

## Results

### Study Population

We included data on 117 493 children (ABIS, *n* = 16 223; MoBa, *n* = 101 270). During 2 024 299 person-years (PYR) of follow-up, 451 participants were diagnosed with IBD (CD = 182, UC = 152, and IBD-U = 117, Supplementary Table S5). Mean follow-up was 22.3 years in ABIS and 16.4 years in MoBa. Reflecting the younger age of the MoBa participants compared to ABIS, the incidence rates for IBD were 20 per 100 000 PYR in MoBa and 31 per 100 000 in ABIS. While maternal education level was higher in MoBa than in ABIS, other baseline characteristics were similar across the cohorts (Table 1).

### Infections in Pregnancy

**Figure 2.**
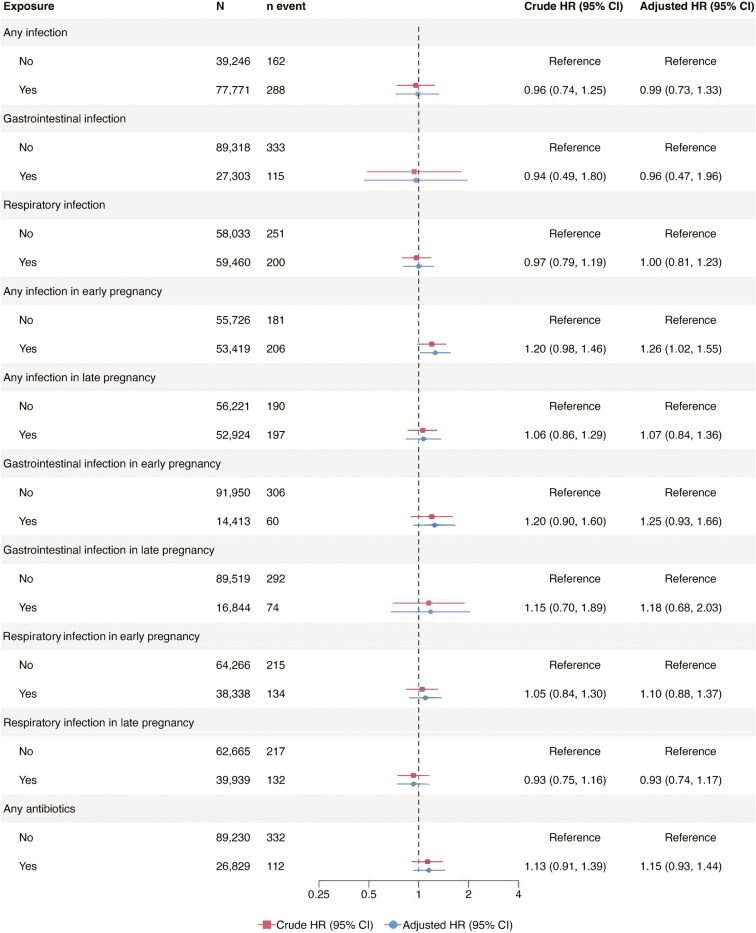
Pooled hazard ratios for maternal infections and antibiotic use in pregnancy and offspring’s risk of inflammatory bowel disease. The timing of infections was analyzed using data from children with information on maternal exposure to infections and the specific time of exposure in pregnancy (any infection/respiratory infection, *n* = 109 145; gastrointestinal infections, *n* = 106 363).

**Figure 3.**
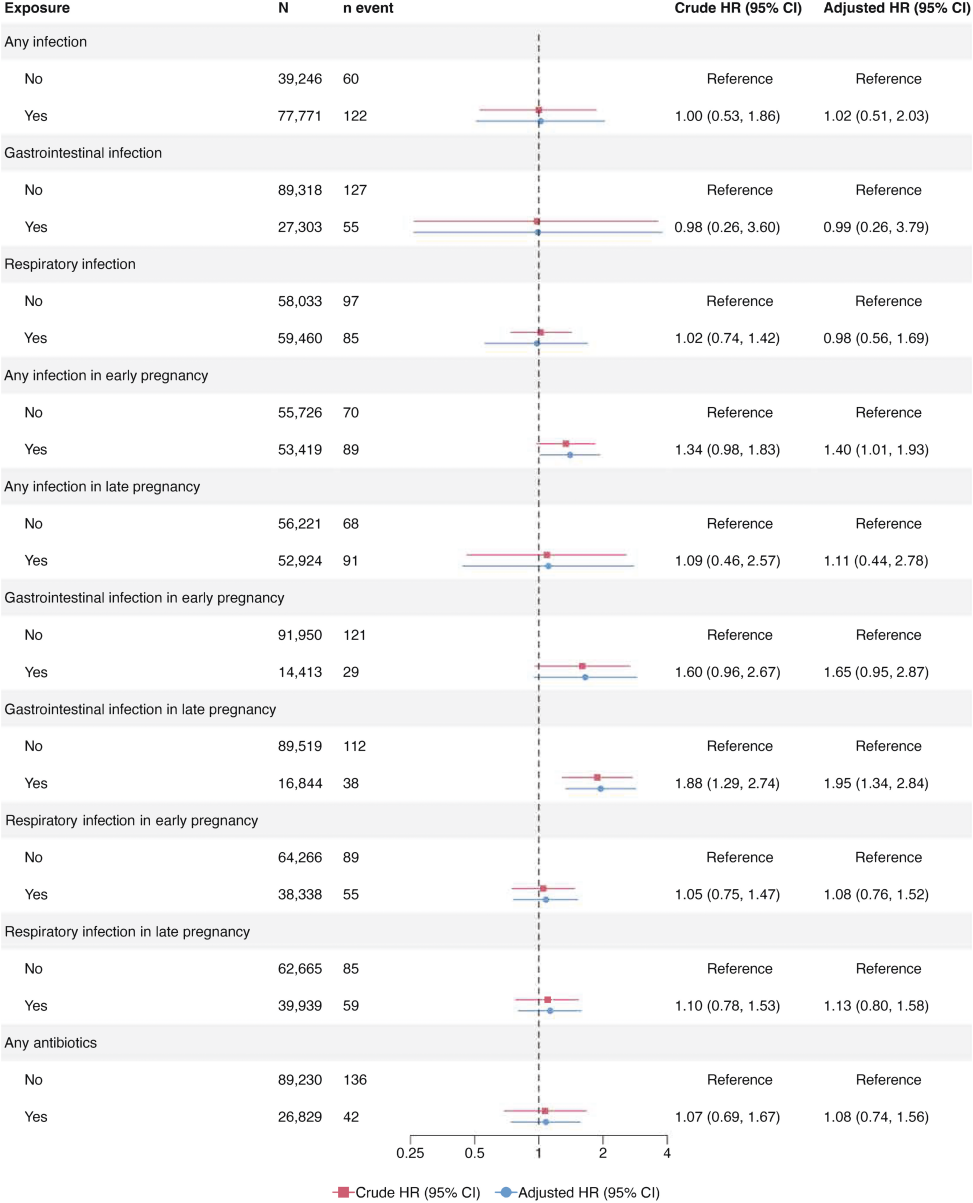
Pooled hazard ratios for maternal infections and antibiotic use in pregnancy and offspring’s risk of Crohn’s disease. The timing of infections was analyzed using data from children with information on maternal exposure to infections and the specific time of exposure in pregnancy (any infection/respiratory infection, *n* = 109 145; gastrointestinal infections, *n* = 106 363).

**Figure 4.**
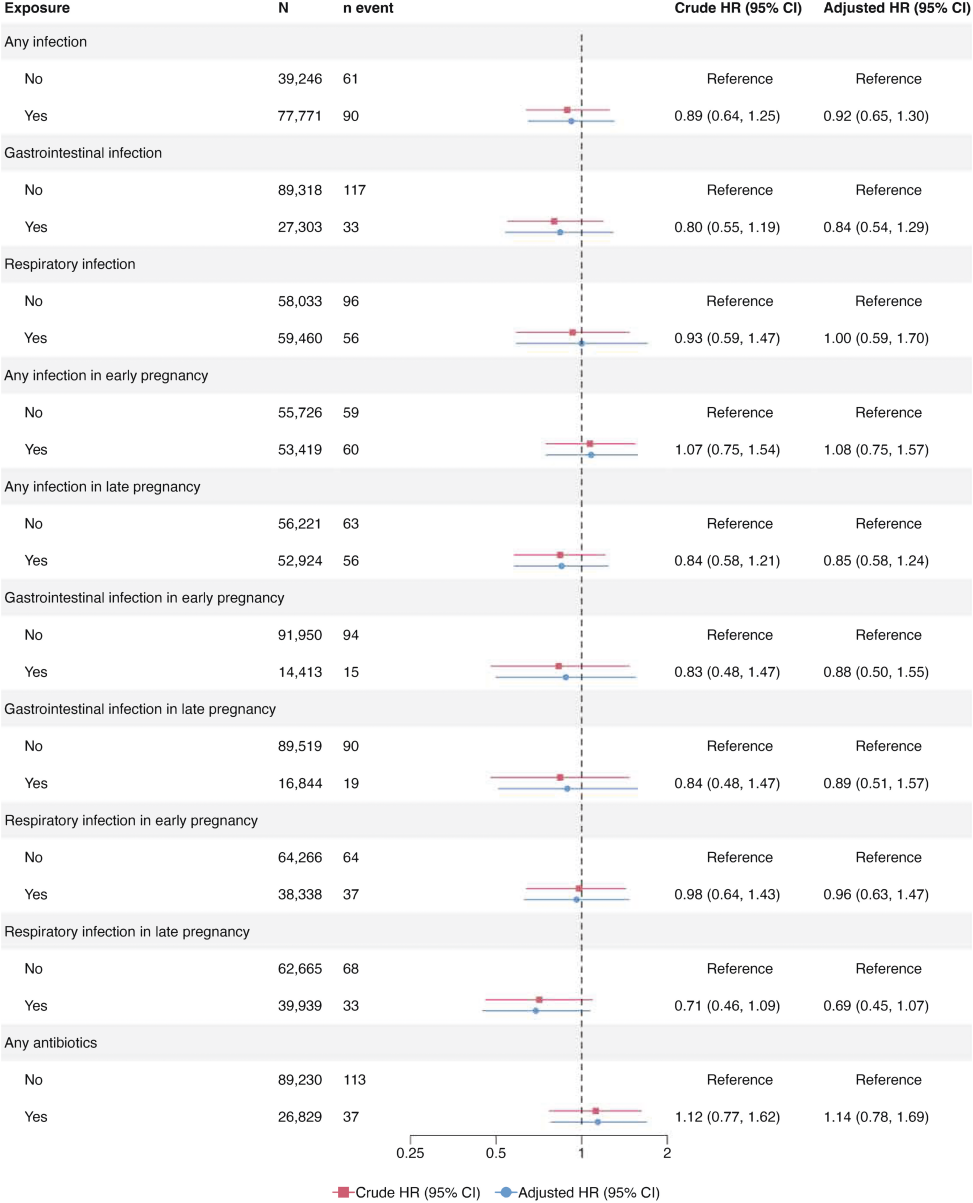
Pooled hazard ratios for maternal infections and antibiotic use in pregnancy and offspring’s risk of ulcerative colitis. The timing of infections was analyzed using data from children with information on maternal exposure to infections and the specific time of exposure in pregnancy (any infection/respiratory infection, *n* = 109 145; gastrointestinal infections, *n* = 106 363).

A total of 77 771 mothers in ABIS and MoBa had available data on any infection in pregnancy (Figure 2). Any versus no maternal infection in pregnancy yielded a pooled aHR of 0.99 for offspring IBD (95% CI = 0.73-1.33; Figure 2), with similar estimates for CD and UC risk (pooled aHR 1.02 [95% CI = 0.51-2.03] and 0.92 [95% CI = 0.65-1.30], respectively; Figures 3-4). Estimates were largely consistent across the 2 cohorts (Supplementary Tables S6-S8). A GI in pregnancy was reported by 23% of all mothers in the cohorts (*n* = 27 303; Figure 2). Pooled analyses revealed no significant associations between any versus no maternal GI in pregnancy and offspring’s risk of IBD (pooled aHR 0.96 [95% CI = 0.47-1.96]; Figure 2), CD or UC (Figures 3-4). The findings in the cohort-specific analyses were similar to the pooled analyses (Supplementary Tables S6-S8). The reported maternal RI during pregnancy observed in 51% of the participants, did not show any association with the risk of IBD, CD, and UC in offspring (Figures 2-4).

### Timing of Infection in Pregnancy

Overall, 109 145 children had data on the timing of any infection and RI in pregnancy, and 106 363 children had data on the timing of maternal GI in pregnancy. Analysis of the pooled data showed that any versus no maternal infection in early pregnancy was associated with an increased risk of offspring IBD (aHR 1.26; [95% CI = 1.02-1.55]) and CD (aHR 1.40; [95% CI = 1.01-1.93], Figures 2-3). Pooled aHR for UC was 1.08 (95% CI = 0.75-1.57; Figure 4). Offspring risk of IBD and its subtypes was not associated with any infection in late pregnancy (Figures 2-4 [pooled analyses] and Supplementary Tables S6-S8 [cohort-specific analyses]).

Comparison of any versus no maternal GI in early pregnancy showed no association with the risk of IBD in offspring (Figures 2-4). In contrast, having any versus no GI in late pregnancy was associated with an almost 2-fold risk of CD in offspring (pooled aHR 1.95; [95% CI = 1.34-2.84]; Figure 3), with similar estimates in MoBa, but not in ABIS (Supplementary Table S7). Pooled aHRs for any versus no GI in late pregnancy were 1.18 (95% CI = 0.68-2.03; Figure 2) for offspring IBD and 0.89 (95% CI = 0.51-1.57) for offspring UC. Maternal RI in early or late pregnancy was not linked to the risk of IBD, CD, and UC in offspring (Figures 2-4).

### Antibiotics in Pregnancy

Some 26 829 mothers reported any antibiotic use in pregnancy. Pooled aHRs of any versus no antibiotic use in pregnancy were 1.15 (95% CI = 0.93-1.44; Figure 2) for IBD, 1.08 (95% CI = 0.74-1.56; Figure 3) for CD, and 1.14 (95% CI = 0.78-1.69; Figure 4) for UC. Cohort-specific analyses yielded no association between antibiotic exposure in pregnancy and offspring’s risk of IBD, CD, and UC (Supplementary Tables S6-S8).

### Sensitivity Analyses

The significant associations were additionally adjusted for (1) maternal antibiotic use in pregnancy and (2) full breastfeeding duration of the child (Supplementary Tables S9-S10). These sensitivity analyses resulted in essentially unchanged HRs, except for the association between GI in late pregnancy and offspring’s risk of CD, which did not remain significant when adjusting for full breastfeeding duration (Supplementary Table S10). For the associations between maternal infections in pregnancy and the risk of IBD and CD in offspring, the effects mediated by the child’s exposure to infections or antibiotic use were close to null (Supplementary Table S11). While insignificant, the largest proportion of mediation observed was 10% (95% CI = −12% to 30%) of the effect of maternal infection on CD risk. This mediation was attributed to an increased frequency of infections in the child (Supplementary Table S11). In sensitivity analyses restricted to childhood-onset IBD (<18 years), excluding VEO-IBD events (<6 years), and analyses adjusted for delivery mode estimates closely mirrored our main analyses (Supplementary Tables S12-S14).

## Discussion

In this study of 2 Scandinavian birth cohorts, we observed that having any versus no infection in the early stages of pregnancy was associated with a modestly increased risk of IBD development in the child, particularly CD. Exposure to GI in late pregnancy was associated with an almost 2-fold risk of subsequent CD in the child, but did not remain significant when adjusted for breastfeeding duration. No association was observed between any maternal infection (GI or RI) at any time in pregnancy, antibiotic use in pregnancy, and the risk of IBD in offspring.

While previous research has shown an association of infections in pregnancy with other pediatric immune-mediated diseases,^22,23^ the few studies conducted on this topic have found no link between maternal infections and the child’s later risk of IBD.^5,9^ However, these studies did not examine the temporal occurrence of infections but rather investigated maternal infection during any stage of pregnancy. In this study, we found infection in early pregnancy and GI in late pregnancy to be associated with the risk of IBD in offspring. Mediation analyses suggested our findings were not mediated by the child’s own infection or early-life antibiotic exposure. These results may indicate that timing and type of infections in pregnancy are important and confer distinct risks, possibly due to different mechanisms for these exposures.

Infections in early pregnancy might affect the development of the fetus’ immune system as the fetal immune system is suggested to start developing as early as GW 12,^17^ and the innate and adaptive immune system is present by GW 16.^16^ The association found for GIs in late pregnancy and offspring CD risk may be explained by the metabolic and immunological alterations occurring in the mother, particularly during the second and third trimesters of pregnancy, which include significant modifications in her gut microbiome.^16,24^ It is possible to hypothesize that the mother’s gut microbiota has an impact on the child’s subsequent microbiome^24^ and immune system,^25^ particularly through vaginal birth. However, we acknowledge that this study did not correct for multiple testing and was unable to distinguish between subtype-specific GI, which has been suggested to influence the risk of CD and UC in adults.^26,27^ In a subset of children with available data on breastfeeding, our analyses of late GI in pregnancy and offspring CD did not remain significant after adjusting for full breastfeeding duration. Hence, the associations noted between early infection and late GIs in pregnancy and IBD risk in offspring warrant a careful interpretation and consideration of alternative explanations for our findings.

Evidence consistently indicates that antibiotic use in children and adults increases the risk of later IBD,^28^ potentially through alterations in the gut microbiome.^29^ Encouragingly, we found no evidence of a link between antibiotics during pregnancy and the risk of IBD in offspring. While a Swedish cohort study of over 800 000 study participants found that children exposed to antibiotics in pregnancy had an increased risk of VEO-IBD,^7^ most other studies have found no such association,^5,6,10^ except for more extreme levels of antibiotic exposure in pregnancy (≥3 courses).^6^ The discrepancy between our findings and those of others may be due to differences in the size of the study population, available data sources, ie, register-based versus self-reported data on antibiotics and that we were not able to explore the cumulative antibiotic exposure. Because of the relatively low numbers of CD and UC events with corresponding wide 95% CIs, we acknowledge that our use of self-reported data on antibiotics may be more prone to misclassification and an increased risk of type 2 error, ie, to accept a false null hypothesis erroneously. The available data also prevented us from investigating the number of antibiotic courses in pregnancy and the specific types of antibiotics used.

We used data on a sample of >117 000 children, among which 451 developed IBD during the follow-up. Unlike previous investigations,^5,9^ we could use prospectively collected data to account for infection and antibiotic use in pregnancy. Through linkage to nationwide health registers,^14,30^ we used an IBD algorithm that demonstrated a positive predictive value of 93% for IBD.^20,21^ To address potential confounding, adjustments were made for parental IBD, smoking habits, education level, maternal antibiotic use, and delivery mode. We examined the mediating effects using the child’s frequency of early-life infection and antibiotic use. This study did not include data on antibiotics from prescription registries. However, the prevalence of antibiotic use in pregnancy is similar to previous studies using data from the Swedish and Norwegian prescription registries.^7,10^ Our exposure definitions are similar to previous ABIS and MoBa data that assessed exposure to infections and antibiotics in pregnancy and the risk of non-communicable diseases in children.^15,22,31,32^ Although there is a greater likelihood of misclassification in self-reported data on infectious diseases and antibiotic use, the study’s prospective design ensures that misclassification will not result in spurious associations but may introduce a bias in our estimates toward null. While our questionnaires capture any infection, register-based data tends to identify more severe infections requiring hospital-based care, which may be more susceptible to selection bias and raise concerns about generalizability. Our investigation did not allow for the examination of infection severity and its association with IBD risk, nor did it permit the analysis of more specific subtypes of infections in relation to IBD risk, eg, viral and bacterial infections.

The study was conducted in 2 countries with high socioeconomic status and followed participants from childhood to young adulthood. Hence, the extent to which our results apply to other settings and IBD development in older adults remains unknown. The participation rate of MoBa was 41%, and the women in this cohort were older, better educated, and less often smoked during pregnancy^33^ than a representative sample of all Norwegian pregnant women.^34^ However, this has previously been shown not to affect the exposure-outcome associations in this cohort.^35^

In conclusion, this study of 2 Scandinavian birth cohorts revealed that maternal infection in early pregnancy and GI in late pregnancy were linked to an increased risk of IBD in offspring, specifically CD, regardless of the mother’s and child’s exposure to antibiotics. However, when adjusting for breastfeeding duration, GI in late pregnancy was not associated with IBD risk. If future studies confirm these novel findings, they may offer valuable insights into the pathogenesis of IBD.

## Supplementary Data

Supplementary data is available at *Inflammatory Bowel Diseases* online.

izae209_suppl_Supplementary_Material

## Data Availability

Due to Swedish and Norwegian legal and ethical regulations, no additional data are available.
