# Supplementary material for: Association Between Maternal Infections in Pregnancy and the Risk of Inflammatory Bowel Disease in the Offspring: Findings From Two Scandinavian Birth Cohorts
Source: Inflamm Bowel Dis. 2024 Sep 9;31(7):1761–71. doi: 10.1093/ibd/izae209 (PMC12235132; doi:10.1093/ibd/izae209)
Supplement: izae209_suppl_Supplementary_Material [file izae209_suppl_supplementary_material.docx]

**SUPPLEMENTARY TO**

*Guo et al. Association between maternal infections in pregnancy and the risk of inflammatory bowel disease in the offspring: Findings from two Scandinavian birth cohorts*

TABLE OF CONTENT

[SUPPLEMENTARY TABLES 3](#_Toc174897486)

[**Supplementary Table 1.** Previous literature 3](#_Toc174897487)

[**Supplementary Table 2.** Data sources of variables used as exposures and covariates^a^ 5](#_Toc174897488)

[**Supplementary Table 3.** Definition of maternal exposure to infections and antibiotics during pregnancy in ABIS and MoBa 6](#_Toc174897489)

[**Supplemental Table 4.** International Statistical Classification of Diseases and Related Health Problems, Tenth Revision (ICD-10) codes for inflammatory bowel disease^a^ 9](#_Toc174897490)

[**Supplemental Table 5.** Number of events, person-years of follow-up, incidence rates and cumulative incidences of inflammatory bowel disease and subtypes in ABIS and MoBa 10](#_Toc174897491)

[**Supplementary Table 6.** Maternal infections and antibiotic use in pregnancy and offspring’s risk of inflammatory bowel disease in ABIS and MoBa^a^ 11](#_Toc174897492)

[**Supplementary Table 7.** Maternal infections and antibiotic use in pregnancy and offspring’s risk of Crohn’s disease in ABIS and MoBa^a^ 13](#_Toc174897493)

[**Supplementary Table 8.** Maternal infections and antibiotic use in pregnancy and offspring’s risk of ulcerative colitis in ABIS and MoBa^a^ 15](#_Toc174897494)

[**Supplementary Table 9.** Any infection in early pregnancy and risk of inflammatory bowel disease in offspring. Sensitivity analyses additionally adjusted for maternal antibiotics in pregnancy and full breastfeeding duration 17](#_Toc174897495)

[**Supplementary Table 10.** Any infection in early pregnancy and gastrointestinal infection in late pregnancy and risk of Crohn’s disease in offspring. Sensitivity analyses additionally adjusted for maternal antibiotics in pregnancy, and full breastfeeding duration 18](#_Toc174897496)

[**Supplementary Table 11.** The effect of maternal infections on the offspring’s risk of inflammatory bowel disease and Crohn’s disease mediated through the child’s infections and treatment with antibiotics by 12 months of age 19](#_Toc174897497)

[**Supplementary Table 12.** Pooled hazard ratios for childhood-onset inflammatory bowel disease (age <18 years, 21](#_Toc174897498)

[**Supplementary Table 13.** Pooled hazard ratios for inflammatory bowel disease diagnosis excluding events of very-early-onset IBD 22](#_Toc174897499)

[**Supplementary Table 14.** Pooled hazard ratios for maternal infections and antibiotic use and risk of inflammatory bowel disease in offspring additionally adjusted for delivery mode 23](#_Toc174897500)

[SUPPLEMENTARY FIGURES 24](#_Toc174897501)

[**Supplementary Figure 1.** Directed Acyclic Graph^9^ depicting exposures (green), outcome (blue), confounding variables (red) and external factors only linked to IBD (grey) used in main analyses. Previous studies suggest an influencing effect from parental IBD,^10^ origin,^11^ and maternal education level,^12^ and smoking in pregnancy.^13^ 24](#_Toc174897502)

[**Supplementary Figure 2.** Directed Acyclic Graph^9^ depicting exposures (green), outcome (blue), confounding variables (red), external factors only linked to IBD (grey), and mediators (orange) used in sensitivity analyses. Previous studies suggest an influencing effect from parental IBD,^10^ origin,^11^ maternal education level,^12^ smoking in pregnancy,^13^ child’s early-life infections and antibiotic use,^14^ and breastfeeding.^15^ 25](#_Toc174897503)

[REFERENCES 26](#_Toc174897504)

**SUPPLEMENTARY TABLES**

**Supplementary Table 1.** Previous literature investigating maternal infections and antibiotics in pregnancy and offspring IBD

| First author, year (country), study design | Study population | Exposure | Main Findings |
| --- | --- | --- | --- |
| Hutfless, 2012 (USA), case-control study ^1^ | 189 IBD cases aged <18years; 3,080 matched controls | Maternal infection defined by ICD-9-CM codes from inpatient and outpatient records. | - Only maternal respiratory infections were examined.  - Maternal respiratory infection in pregnancy was not associated with an increase oods ratio (OR) for IBD compared to healthy controls (IBD, fully adjusted (a)OR 2.0 [95% CI 1.0–4.0]; CD, aOR 2.0 [95% CI 0.5–7.6]; UC, aOR 1.9 [95% CI 0.7–5.5]). |
| Bernstein, 2017 (Canada), case-control study ^2^ | 2,487 IBD cases (1,671 linked mother-child dyads); 10,488 matched controls;  1,740 siblings | Antibiotics defined by ATC code J01  Infection defined by ICD-8 and ICD-9 from inpatient and hospitalized based records. | - Compared to healthy controls, children with IBD were not more likely to have mothers with infection in pregnancy (OR 0.96 [0.84–1.09]) |
|  |  |  | - Did not detect any difference in the prevalence of maternal antibiotic exposure during the pregnancies of IBD cases (32.3%), matched controls (36.5%, P = 0.75), and their unaffected siblings (30.1%, P = 0.07). |
| Örtkvist, 2019 (Sweden), cohort study ^3^ | Cohort of 827,239 children out of whom  51 had VEO-IBD (diagnosed <6y) | Antibiotics defined by ATC J01A-J01X from the Swedish Prescribed Drug Register | - Children exposed vs not exposed to systemic antibiotics in pregnancy had an increased risk of IBD (Adjusted (a)HR 1.93, 95% CI 1.06-3.50). Corresponding aHRs were 2.48 (95% CI 1.01-6.08) for CD and 1.25 (95% CI 0.47-3.26) for UC.  - aHR for children exposed to any antibiotics were 1.93 (95% CI 1.06-3.50) for IBD, 2.48 (95% CI 1.01-6.08) for CD and 1.25 (95% CI 0.47-3.26) for UC.  - aHR for children exposed to PcV were 2.15 (95% CI 1.02-4.56) for IBD, 2.85 (95% CI 0.96-8.45) for CD and 1.68 (95% CI 0.51-5.97) for UC. |
| Torres, 2023, (Denmark), case-control study ^4^ | 1,732 IBD cases;  2,447 non-IBD controls | Antibiotics defined by ATC J01 divided into narrow and broad-spectrum from the National Prescription Registry | - Maternal exposure to antibiotics in pregnancy was not associated with offspring’s risk of paediatric IBD in within-family analysis (aOR 0.75 [95% CI 0.32-1.75]) or across-family analysis (aOR 0.81 [95% CI 0.56-1.17]). |
| Agrawal, 2023 (Denmark), cohort study ^5^ | Cohort of 416,833 mother-child dyads | Antibiotics defined by ATC J01 divided into narrow and broad-spectrum from the Danish National Prescription Register | - Overall, maternal antibiotic use vs no use in pregnancy was not associated with offspring IBD, UC or CD risk (aHR 1.01, 95% CI 0.91 to 1.12; aHR 1.03, 95% CI 0.89 to 1.2; and aHR 0.99, 95% CI 0.86 to 1.14, respectively). - Children to mothers exposed to >3 courses, vs none, were associated with risk of IBD (aHR 1.29, 95% CI 1.03, 1.62) and UC (aHR 1.45, 95%CI 1.06 to 2.00) but not CD (aHR 1.15, 95%CI 0.83 to 1.60).  - There was no impact of the trimester of antibiotic exposure or narrow-spectrum versus broad-spectrum antibiotics. |
| Andersen, 2024 (Norway), cohort study^6^ | 797 IBD cases <18 years; 536,022 controls | Antibiotics defined by ATC J01 dispensed within 2 first years of life and to the mothers during pregnancy from the Norwegian Prescription Database and Medical Birth Registry of Norway | - Adjusted OR for developing paediatric IBD and exposed vs non-exposed to antibiotics <2 years of age was 1.33 (95 CI 1.15-1.53)  - An increased risk was seen for those with >2 antibiotic courses (aOR 1.47 [95% CI 1.25-1.73]), with higher estimates for broad-spectrum antibiotics (aOR 2.57 [95% CI 1.82-3.63]).  - No association was found between overall antibiotics in pregnancy and offspring paediatric IBD. |

ATC code, Anatomical Therapeutic Chemical codes; CD, Crohn’s disease; HR, hazard ratio; IBD, inflammatory bowel disease, OR, odds ratio; PcV, phenoxymethylpenicillin; UC, ulcerative colitis; VEO-IBD, very early onset inflammatory bowel disease

## **Supplementary Table 2.** Data sources of variables used as exposures and covariates^a^

|  | ABIS^b^ | MoBa^c^ |
| --- | --- | --- |
| **Exposures** |  |  |
| Any infection | Birth questionnaire | 15 GW, 30 GW, 6 months questionnaires |
| Early infection^c^ | Birth questionnaire | 15 GW, 30 GW, 6 months questionnaires |
| Late infection^d^ | Birth questionnaire | 15 GW, 30 GW, 6 months questionnaires |
| Gastrointestinal infection | Birth questionnaire | 15 GW, 30 GW, 6 months questionnaires |
| Early gastrointestinal infection^c^ | Birth questionnaire | 15 GW, 30 GW, 6 months questionnaires |
| Late gastrointestinal infection^d^ | Birth questionnaire | 15 GW, 30 GW, 6 months questionnaires |
| Respiratory infection | Birth questionnaire | 15 GW, 30 GW, 6 months questionnaires |
| Early respiratory infection^c^ | Birth questionnaire | 15 GW, 30 GW, 6 months questionnaires |
| Late respiratory infection^d^ | Birth questionnaire | 15 GW, 30 GW, 6 months questionnaires |
| Antibiotic use | Birth questionnaire | 15 GW, 30 GW, 6 months questionnaires |
| **Covariates** |  |  |
| Child’s sex | Birth questionnaire | Medical Birth Registry of Norway |
| Parental origin^f^ | Birth questionnaire | 15 GW questionnaire |
| Maternal education level^g^ | Birth questionnaire | 15 GW questionnaire |
| Parental IBD^h^ | Birth questionnaire | Norwegian Patient Registry |
| Maternal smoking in pregnancy | Birth questionnaire | 15 GW, 30 GW, 6 months questionnaires |
| Child’s infection 0-12 months^i^ | 12 month questionnaire | 18 month questionnaire |
| Child’s antibiotic use 0-12 months^i^ | 12 month questionnaire | 18 month questionnaire |
| Full breastfeeding^j^ | 12 month questionnaire | 6 and 18 month questionnaire |
| Delivery mode^k^ | Birth questionnaire | Medical Birth Registry of Norway |

^a^Data were only captured at one time point, except for maternal smoking (MoBa).

^b^In ABIS, the mothers were asked to report types of infections in questionnaires administered at birth which covered all of pregnancy.

^c^In MoBa, the mothers were asked to report types of infections in questionnaires administered at gestational weeks 15, 30 and 6 months after the pregnancy which covered gestational weeks 15-40.

^d^ Reported at the child’s birth (ABIS) or gestational weeks 15 and 30 and 6 months after the child’s birth (MoBa). Early pregnancy was defined as the first <4 months of pregnancy (ABIS) and gestational week <16 (MoBa). Late pregnancy was defined as the last >5 months of pregnancy (ABIS) and gestational week >17 (MoBa).

^f^Mother’s native language (MoBa)/parent’s country of birth (ABIS).

^g^Education at time of birth.

^h^Defined as >1 parent with IBD and captured at birth/in pregnancy.

^i^Categorised as exposed or unexposed.

^j^<4 months, 4-6 months, >6 months

^k^vaginal, cesarean section

ABIS, All Babies in Southeast Sweden; GW, gestational week; IBD, inflammatory bowel disease; MoBa, The Norwegian Mother, Father and Child Cohort Study.

##

## **Supplementary Table 3.** Definition of maternal exposure to infections and antibiotics during pregnancy in ABIS and MoBa

|  | ABIS | MoBa |
| --- | --- | --- |
| **Any infection in pregnancy (yes vs no)** | *Birth questionnaire* Q108. Did you have any gastric flue (diarrhea, fever, vomiting) during pregnancy?  1. Yes = Yes 2. No = No  3. Don’t know = Missing  Q109. Did you have any other infections during pregnancy?  1. Yes = Yes 2. No = No  3. Don’t know = Missing | *GW15 questionnaire* Q38. Have you experienced any of the following illnesses during this pregnancy?  11. Diarrhea/gastric flu 16. Fever with rash 17. Fever over 38.5 C 18. Common cold 19. Throat infection 20. Sinusitis/ear infection 21. Influenza 22. Pneumonia/bronchitis  Q39. Do you have /had any of the following illnesses? 41. Kidney infection/pyelonephritis 42. Urinary tract infection/cystitis  *GW30 questionnaire*  Q52. Do you have or have you had any of the following illnesses after GW 30?  10. Diarrhea/gastric flu 14. Common cold 15. Throat infection 16. Sinusitis/ear infection 17. Influenza 18. Pneumonia/bronchitis 19. Other cough  *6-month questionnaire (covering GW30-birth)*  Q53. Have you had any of the following illnesses during pregnancy since GW 30?  5. Cystitis 7. Diarrhea/vomiting 9. Common cold/influenza 10. Sore throat/sinusitis/ear infection 11. Pneumonia/bronchitis 18. Fever |
| **Timing of any infections in pregnancy**  **(any vs no infection in early pregnancy / any vs no infection in late pregnancy)** | *Birth questionnaire* Q108. Did you have any gastric flue (diarrhea, fever, vomiting) during pregnancy? If yes, which pregnancy month?  <4 months = Early pregnancy  >5 months = Late pregnancy  Q109. Did you have any other infections during pregnancy? If yes, which pregnancy month?  <4 months = Early pregnancy  >5 months = Late pregnancy | *GW15 questionnaire*  *GW30 questionnaire*  *6-month questionnaire (covering GW30-birth)*  Questions and answers used for  Any infection (yes vs no) GW <16 = Early pregnancy  GW ≥17 = Late pregnancy |
| **Gastrointestinal infection in pregnancy (yes vs no)** | Q108. Did you have any gastric flue (diarrhea, fever, vomiting) during pregnancy? 1. Yes = Yes 2. No = No  3. Don’t know = Missing | *GW15 questionnaire*  Q38. Have you experienced any of the following illnesses during this pregnancy? 11. Diarrhea/gastric flu  *GW30 questionnaire*  Q52. Do you have or have you had any of the following illnesses after GW30?  10. Diarrhea/gastric flu  *6-month questionnaire (covering GW30-birth)*  Q53. Have you had any of the following illnesses during pregnancy since GW 30? 7. Diarrhea/vomiting |
| **Timing of gastrointestinal infection in pregnancy (any vs no infection in early pregnancy / any vs no infection in late pregnancy)** | *Birth questionnaire* Q108. Did you have any gastric flue (diarrhea, fever, vomiting) during pregnancy? If yes, which pregnancy month?  <4 months = Early pregnancy  >5 months = Late pregnancy | *GW15 questionnaire*  *GW30 questionnaire*  *6-month questionnaire (covering GW30-birth)*  Questions and answers used for  Gastrointestinal infection (yes vs no) GW <16 = Early pregnancy  GW ≥17 = Late pregnancy |
| **Respiratory tract infection in pregnancy (yes vs no)** | *Birth questionnaire*  Q109. Did you have any other infections during pregnancy? If yes, what? (open answer)  “Yes” is defined as any reported respiratory tract infections, e.g., common cold, throat infection/tonsillitis, pneumonia/bronchitis, sinusitis/ear infection | *GW15 questionnaire*  Q38. Have you experienced any of the following illnesses during this pregnancy? 18. Common cold 19. Throat infection 20. Sinusitis/ear infection 22. Pneumonia/bronchitis  *GW30 questionnaire*  Q52. Do you have or have you had any of the following illnesses after GW30?  14. Common cold 15. Throat infection 16. Sinusitis/ear infection 18. Pneumonia/bronchitis  *6-month questionnaire (covering GW30-birth)*  Q53. Have you had any of the following illnesses during pregnancy since GW 30?  9. Common cold/influenza 10. Sore throat/ sinusitis/ear infection 11. Pneumonia/bronchitis |
| **Timing of respiratory infection in pregnancy (any vs no infection in early pregnancy / any vs no infection in late pregnancy)** | Q109. Did you have any other infections during pregnancy? If yes, which pregnancy month? Defined as respiratory tract infection (yes vs no)  <4 months = Early pregnancy  >5 months = Late pregnancy | *GW15 questionnaire*  *GW30 questionnaire*  *6-month questionnaire (covering GW30-birth)*  Questions and answers used for  Respiratory tract infection (yes vs no) GW <16 = Early pregnancy  GW ≥17 = Late pregnancy |
| **Any antibiotics in pregnancy (yes vs no)** | Q17a. Do you take any medications during pregnancy? Antibiotics 1. Yes = Yes 2. No = No  3. Don’t know = Missing | *GW15 questionnaire* Q38. Have you experienced any of the following illnesses or problems during this pregnancy? If you have used medication in connection  with these problems give the name of the medicine. 11. Diarrhea/gastric flu 16. Fever with rash 17. Fever over 38.5 C 18. Common cold 19. Throat infection20. Sinusitis/ear infection 21. Influenza 22. Pneumonia/bronchitis  Q39. Do you have or have you had any of the following illnesses or health problems? If you have taken medication (tablets, mixtures, suppositories,  inhalers, creams, etc.) in conjunction with the illness or health problem, give the name(s) of the medication(s) and when you took them.  41. Kidney infection/pyelonephritis 42. Urinary tract infections/cystitis  *GW30 questionnaire*  Q52. Do you have or have you had any of the following illnesses or problems after the 13th week of pregnancy? If you have used tablets,  mixtures, suppositories, inhalers, creams, etc. in connection with the illness or problem, give the name(s) of the medication(s), when and  how long you took them.  10. Diarrhea/gastric flu 14. Common cold 15. Throat infection 16. Sinusitis/ear infection 17. Influenza 18. Pneumonia/bronchitis 19. Other cough  *6-month questionnaire (covering GW30-birth)*  Q53. Have you had any of the following problems/illnesses since you completed the previous questionnaire (GW 30)? If yes, are you taking or have you  taken medication for these problems? 7. Diarrhea/vomiting 9. Common cold/influenza 10. Sore throat/sinusitis/ear infection 11. Pneumonia/bronchitis 18. Fever |

## **Supplemental Table 4.** International Statistical Classification of Diseases and Related Health Problems, Tenth Revision (ICD-10) codes for inflammatory bowel disease^a^

| Disease | ICD-10 (1997-) | Description |
| --- | --- | --- |
| Inflammatory bowel disease | K52.3, CD + UC or mixed diagnosis of CD, UC and IBD-U (K52.3)^b^ | IBD diagnosis required at least two inpatient or non-primary outpatient care diagnoses. Patients who shifted in IBD subtype diagnosis during the last five years were classified according to the most recent diagnosis. Those with a mix of CD, UC and IBD-U codes during the last five years of follow-up were classified as  (“any, non-specific”) IBD-U. |
| Crohn’s disease | K50 |  |
| Ulcerative colitis | K51 |  |

^a^Based on validated register-based IBD definition.^7, 8^

^b^IBD-U was not measured as a separate outcome.

CD, Crohn’s disease; IBD, inflammatory bowel disease; IBD-U, inflammatory bowel disease unclassified; UC, ulcerative colitis.

## **Supplemental Table 5.** Number of events, person-years of follow-up, incidence rates and cumulative incidences of inflammatory bowel disease and subtypes in ABIS and MoBa

|  | n events | PYR of follow-up | Incidence rate per 100,000 PYR (95%CI) | Cumulative incidence (%) at the end of follow-up^a^ | Cumulative incidence (%) by <15 years of age^b^ |
| --- | --- | --- | --- | --- | --- |
| **ABIS** |  |  |  |  |  |
| IBD^c^ | 113 | 360,655 | 31.3 (25.8, 37.7) | 0.70 (0.57, 0.84) | 0.17 (0.11, 0.25) |
| CD | 40 | 361,032 | 11.1 (7.9, 15.1) | 0.25 (0.18, 0.34) | 0.08 (0.04, 0.14) |
| UC | 57 | 360,963 | 15.8 (12.0, 20.5) | 0.35 (0.27, 0.45) | 0.08 (0.04, 0.14) |
| **MoBa** |  |  |  |  |  |
| IBD^c^ | 338 | 1,663,644 | 20.3 (18.2, 22.6) | 0.33 (0.30, 0.37) | 0.23 (0.20, 0.26) |
| CD | 142 | 1,664,544 | 8.5 (7.2, 10.1) | 0.14 (0.12, 0.17) | 0.10 (0.08, 0.13) |
| UC | 95 | 1,664,718 | 5.7 (4.6, 7.0) | 0.09 (0.08, 0.11) | 0.05 (0.04, 0.07) |

^a^End of follow-up was December 31, 2020 (ABIS) and December 31, 2021 (MoBa). Mean age at the end of follow-up was 22.2 years in ABIS and 16.4 years in MoBa.

^b^Due to the different follow-up times in ABIS and MoBa, we restricted this to IBD events with onset before 15

years of age.

^c^Includes CD, UC and IBD-U [not measured as a separate outcome].

ABIS, All Babies in Southeast Sweden study; CD, Crohn’s disease; CI, confidence interval; IBD, inflammatory bowel disease; IBD-U, IBD-unclassified; MoBa, The Norwegian Mother, Father and Child Cohort Study; PYR, person-year; UC, ulcerative colitis.

## **Supplementary Table 6.** Maternal infections and antibiotic use in pregnancy and offspring’s risk of inflammatory bowel disease in ABIS and MoBa^a^

|  | MoBa | | | | | | ABIS | | | | | |
| --- | --- | --- | --- | --- | --- | --- | --- | --- | --- | --- | --- | --- |
|  | n | n events | HR | 95% CI | aHR^b^ | 95% CI | n | n events | HR | 95% CI | aHR^b^ | 95% CI |
| Any infection |  |  |  |  |  |  |  |  |  |  |  |  |
| No | 31,664 | 102 | — | — | — | — | 7,582 | 60 | — | — | — | — |
| Yes | 69,606 | 236 | 1.07 | 0.84, 1.35 | 1.12 | 0.88, 1.43 | 8,165 | 52 | 0.81 | 0.56, 1.17 | 0.82 | 0.56, 1.19 |
| Gastrointestinal infection |  |  |  |  |  |  |  |  |  |  |  |  |
| No | 79,240 | 251 | — | — | — | — | 10,078 | 82 | — | — | — | — |
| Yes | 22,030 | 87 | 1.28 | 1.00, 1.63 | 1.35 | 1.05, 1.73 | 5,273 | 28 | 0.65 | 0.43, 1.00 | 0.65 | 0.42, 1.00 |
| Respiratory infection |  |  |  |  |  |  |  |  |  |  |  |  |
| No | 43,318 | 150 | — | — | — | — | 14,715 | 101 | — | — | — | — |
| Yes | 57,952 | 188 | 0.95 | 0.76, 1.18 | 0.98 | 0.78, 1.22 | 1,508 | 12 | 1.15 | 0.63, 2.10 | 1.17 | 0.65, 2.12 |
| Any infection in early pregnancy |  |  |  |  |  |  |  |  |  |  |  |  |
| No | 51,766 | 160 | — | — | — | — | 3,960 | 21 | — | — | — | — |
| Yes | 49,504 | 178 | 1.18 | 0.95, 1.46 | 1.24 | 1.00, 1.55 | 3,915 | 28 | 1.35 | 0.77, 2.39 | 1.34 | 0.76, 2.37 |
| Any infection in late pregnancy |  |  |  |  |  |  |  |  |  |  |  |  |
| No | 53,854 | 173 | — | — | — | — | 2,367 | 17 | — | — | — | — |
| Yes | 47,416 | 165 | 1.09 | 0.88, 1.35 | 1.13 | 0.91, 1.41 | 5,508 | 32 | 0.80 | 0.45, 1.45 | 0.80 | 0.45, 1.45 |
| Gastrointestinal infection in early pregnancy |  |  |  |  |  |  |  |  |  |  |  |  |
| No | 89,200 | 293 | — | — | — | — | 2,750 | 13 | — | — | — | — |
| Yes | 12,070 | 45 | 1.17 | 0.86, 1.60 | 1.23 | 0.90, 1.68 | 2,343 | 15 | 1.37 | 0.65, 2.88 | 1.35 | 0.64, 2.83 |
| Gastrointestinal infection in late pregnancy |  |  |  |  |  |  |  |  |  |  |  |  |
| No | 87,630 | 280 | — | — | — | — | 1,889 | 12 | — | — | — | — |
| Yes | 13,640 | 58 | 1.36 | 1.02, 1.80 | 1.43 | 1.07, 1.90 | 3,204 | 16 | 0.78 | 0.37, 1.64 | 0.79 | 0.37, 1.68 |
| Respiratory infection in early pregnancy |  |  |  |  |  |  |  |  |  |  |  |  |
| No | 63,607 | 211 | — | — | — | — | 659 | 4 | — | — | — | — |
| Yes | 37,663 | 127 | 1.03 | 0.83, 1.28 | 1.08 | 0.86, 1.36 | 675 | 7 | 1.71 | 0.50, 5.84 | 1.68 | 0.49, 5.71 |
| Respiratory infection in late pregnancy |  |  |  |  |  |  |  |  |  |  |  |  |
| No | 62,333 | 213 | — | — | — | — | 332 | 4 | — | — | — | — |
| Yes | 38,937 | 125 | 0.95 | 0.76, 1.18 | 0.95 | 0.75, 1.19 | 1,002 | 7 | 0.58 | 0.17, 1.98 | 0.59 | 0.17, 1.98 |
| Any antibiotics |  |  |  |  |  |  |  |  |  |  |  |  |
| No | 77,437 | 250 | — | — | — | — | 11,793 | 82 | — | — | — | — |
| Yes | 23,833 | 88 | 1.12 | 0.88, 1.42 | 1.15 | 0.90, 1.48 | 2,996 | 24 | 1.16 | 0.73, 1.82 | 1.17 | 0.74, 1.84 |

aHR: adjusted hazard ratio; CI, confidence interval; HR, hazard ratio.

^a^Reported at child’s birth (ABIS) or gestational weeks 15 and 30 and at child’s age 6 months (MoBa). Early pregnancy was defined as the first <4 months of pregnancy. Late pregnancy was defined as the last >5 months of pregnancy (ABIS) and gestational week >17 (MoBa) (Supplementary Table 2-3).

^b^Adjusted for the child’s sex, parental inflammatory bowel disease, parental origin, maternal smoking in pregnancy, and maternal education level (Supplementary Table 2).

## **Supplementary Table 7.** Maternal infections and antibiotic use in pregnancy and offspring’s risk of Crohn’s disease in ABIS and MoBa^a^

|  | MoBa | | | | | | ABIS | | | | | |
| --- | --- | --- | --- | --- | --- | --- | --- | --- | --- | --- | --- | --- |
|  | n | n events | HR | 95% CI | aHR^b^ | 95% CI | n | n events | HR | 95% CI | aHR^b^ | 95% CI |
| Any infection |  |  |  |  |  |  |  |  |  |  |  |  |
| No | 31,664 | 37 | — | — | — | — | 7,582 | 23 | — | — | — | — |
| Yes | 69,606 | 105 | 1.31 | 0.90, 1.90 | 1.39 | 0.94, 2.05 | 8,165 | 17 | 0.69 | 0.37, 1.28 | 0.68 | 0.36, 1.28 |
| Gastrointestinal infection |  |  |  |  |  |  |  |  |  |  |  |  |
| No | 79,240 | 95 | — | — | — | — | 10,078 | 32 | — | — | — | — |
| Yes | 22,030 | 47 | 1.82 | 1.28, 2.57 | 1.88 | 1.33, 2.67 | 5,273 | 8 | 0.48 | 0.22, 1.04 | 0.48 | 0.22, 1.03 |
| Respiratory infection |  |  |  |  |  |  |  |  |  |  |  |  |
| No | 43,318 | 59 | — | — | — | — | 14,715 | 38 | — | — | — | — |
| Yes | 57,952 | 83 | 1.06 | 0.76, 1.49 | 1.09 | 0.77, 1.54 | 1,508 | 2 | 0.51 | 0.12, 2.12 | 0.48 | 0.12, 1.97 |
| Any infection in early pregnancy |  |  |  |  |  |  |  |  |  |  |  |  |
| No | 51,766 | 64 | — | — | — | — | 3,960 | 6 | — | — | — | — |
| Yes | 49,504 | 78 | 1.29 | 0.92, 1.80 | 1.35 | 0.96, 1.90 | 3,915 | 11 | 1.86 | 0.69, 5.02 | 1.84 | 0.69, 4.92 |
| Any infection in late pregnancy |  |  |  |  |  |  |  |  |  |  |  |  |
| No | 53,854 | 61 | — | — | — | — | 2,367 | 7 | — | — | — | — |
| Yes | 47,416 | 81 | 1.52 | 1.09, 2.12 | 1.60 | 1.14, 2.25 | 5,508 | 10 | 0.61 | 0.23, 1.61 | 0.61 | 0.23, 1.61 |
| Gastrointestinal infection in early pregnancy | |  |  |  |  |  |  |  |  |  |  |  |
| No | 89,200 | 119 | — | — | — | — | 2,750 | 2 | — | — | — | — |
| Yes | 12,070 | 23 | 1.47 | 0.94, 2.29 | 1.49 | 0.95, 2.32 | 2,343 | 6 | 3.53 | 0.71, 17.46 | 3.53 | 0.76, 16.54 |
| Gastrointestinal infection in late pregnancy |  |  |  |  |  |  |  |  |  |  |  |  |
| No | 87,630 | 109 | — | — | — | — | 1,889 | 3 | — | — | — | — |
| Yes | 13,640 | 33 | 1.98 | 1.34, 2.92 | 2.06 | 1.39, 3.04 | 3,204 | 5 | 0.98 | 0.23, 4.11 | 0.98 | 0.24, 3.98 |
| Respiratory infection in early pregnancy |  |  |  |  |  |  |  |  |  |  |  |  |
| No | 63,607 | 88 | — | — | — | — | 659 | 1 | — | — | — | — |
| Yes | 37,663 | 54 | 1.05 | 0.74, 1.48 | 1.08 | 0.76, 1.53 | 675 | 1 | 0.98 | 0.06, 15.59 | 0.95 | 0.06, 15.08 |
| Respiratory infection in late pregnancy |  |  |  |  |  |  |  |  |  |  |  |  |
| No | 62,333 | 84 | — | — | — | — | 332 | 1 | — | — | — | — |
| Yes | 38,937 | 58 | 1.12 | 0.80, 1.56 | 1.15 | 0.81, 1.62 | 1,002 | 1 | 0.33 | 0.02, 5.29 | 0.33 | 0.02, 5.21 |
| Any antibiotics |  |  |  |  |  |  |  |  |  |  |  |  |
| No | 77,437 | 110 | — | — | — | — | 11,793 | 26 | — | — | — | — |
| Yes | 23,833 | 32 | 0.93 | 0.62, 1.37 | 0.97 | 0.65, 1.44 | 2,996 | 10 | 1.52 | 0.73, 3.14 | 1.50 | 0.72, 3.14 |

aHR: adjusted hazard ratio; CI, confidence interval; HR, hazard ratio.

^a^Reported at child’s birth (ABIS) or gestational weeks 15 and 30 and at child’s age 6 months (MoBa). Early pregnancy was defined as the first <4 months of pregnancy. Late pregnancy was defined as the last >5 months of pregnancy (ABIS) and gestational week >17 (MoBa) (Supplementary Table 2-3).

^b^Adjusted for the child’s sex, parental inflammatory bowel disease, parental origin, maternal smoking in pregnancy, and maternal education level (Supplementary Table 2).

## **Supplementary Table 8.** Maternal infections and antibiotic use in pregnancy and offspring’s risk of ulcerative colitis in ABIS and MoBa^a^

|  | MoBa | | | | | | ABIS | | | | | |
| --- | --- | --- | --- | --- | --- | --- | --- | --- | --- | --- | --- | --- |
|  | n | n events | HR | 95% CI | aHR^b^ | 95% CI | n | n events | HR | 95% CI | aHR^b^ | 95% CI |
| Any infection |  |  |  |  |  |  |  |  |  |  |  |  |
| No | 31,664 | 31 | — | — | — | — | 7,582 | 30 | — | — | — | — |
| Yes | 69,606 | 64 | 0.96 | 0.62, 1.47 | 1.0 | 0.63, 1.57 | 8,165 | 26 | 0.81 | 0.48, 1.37 | 0.84 | 0.50, 1.41 |
| Gastrointestinal infection |  |  |  |  |  |  |  |  |  |  |  |  |
| No | 79,240 | 76 | — | — | — | — | 10,078 | 41 | — | — | — | — |
| Yes | 22,030 | 19 | 0.93 | 0.56, 1.54 | 1.01 | 0.61, 1.69 | 5,273 | 14 | 0.66 | 0.36, 1.20 | 0.65 | 0.35, 1.20 |
| Respiratory infection |  |  |  |  |  |  |  |  |  |  |  |  |
| No | 43,318 | 46 | — | — | — | — | 14,715 | 50 | — | — | — | — |
| Yes | 57,952 | 49 | 0.81 | 0.54, 1.22 | 0.83 | 0.54, 1.27 | 1,508 | 7 | 1.36 | 0.62, 3.00 | 1.47 | 0.68, 3.17 |
| Any infection in early pregnancy |  |  |  |  |  |  |  |  |  |  |  |  |
| No | 51,766 | 47 | — | — | — | — | 3,960 | 12 | — | — | — | — |
| Yes | 49,504 | 48 | 1.09 | 0.73, 1.62 | 1.1 | 0.73, 1.67 | 3,915 | 12 | 1.02 | 0.46, 2.27 | 1.02 | 0.46, 2. 27 |
| Any infection in late pregnancy |  |  |  |  |  |  |  |  |  |  |  |  |
| No | 53,854 | 56 | — | — | — | — | 2,367 | 7 | — | — | — | — |
| Yes | 47,416 | 39 | 0.80 | 0.53, 1.21 | 0.81 | 0.53, 1.24 | 5,508 | 17 | 1.03 | 0.43, 2.50 | 1.02 | 0.42, 2.46 |
| Gastrointestinal infection in early pregnancy | |  |  |  |  |  |  |  |  |  |  |  |
| No | 89,200 | 85 | — | — | — | — | 2,750 | 9 | — | — | — | — |
| Yes | 12,070 | 10 | 0.91 | 0.47, 1.75 | 0.97 | 0.50, 1.87 | 2,343 | 5 | 0. 66 | 0.22, 1.99 | 0.66 | 0.22, 2.00 |
| Gastrointestinal infection in late pregnancy |  |  |  |  |  |  |  |  |  |  |  |  |
| No | 87,630 | 85 | — | — | — | — | 1,889 | 5 | — | — | — | — |
| Yes | 13,640 | 10 | 0.77 | 0.40, 1.49 | 0.84 | 0.44, 1.63 | 3,204 | 9 | 1.04 | 0.35, 3.11 | 1.04 | 0.35, 3.12 |
| Respiratory infection in early pregnancy |  |  |  |  |  |  |  |  |  |  |  |  |
| No | 63,607 | 62 | — | — | — | — | 659 | 2 | — | — | — | — |
| Yes | 37,663 | 33 | 0.92 | 0.61, 1.38 | 0.92 | 0.59, 1.42 | 675 | 4 | 1.96 | 0.36, 10.67 | 1.94 | 0.36, 10.39 |
| Respiratory infection in late pregnancy |  |  |  |  |  |  |  |  |  |  |  |  |
| No | 62,333 | 66 | — | — | — | — | 332 | 2 | — | — | — | — |
| Yes | 38,937 | 29 | 0.71 | 0.46, 1.11 | 0.69 | 0.44, 1.09 | 1,002 | 4 | 0.66 | 0.12, 3.61 | 0.66 | 0.12, 3.56 |
| Any antibiotics |  |  |  |  |  |  |  |  |  |  |  |  |
| No | 77,437 | 69 | — | — | — | — | 11,793 | 44 | — | — | — | — |
| Yes | 23,833 | 26 | 1,18 | 0.76, 1.85 | 1,22 | 0.76, 1.97 | 2,996 | 11 | 0.99 | 0.51, 1.91 | 1.01 | 0.52, 1.95 |

aHR: adjusted hazard ratio; CI, confidence interval; HR, hazard ratio.

^a^Reported at child’s birth (ABIS) or gestational weeks 15 and 30 and at child’s age 6 months (MoBa). Early pregnancy was defined as the first <4 months of pregnancy. Late pregnancy was defined as the last >5 months of pregnancy (ABIS) and gestational week >17 (MoBa) (Supplementary Table 2-3).

^b^Adjusted for the child’s sex, parental inflammatory bowel disease, parental origin, maternal smoking in pregnancy, and maternal education level (Supplementary Table 2).

## **Supplementary Table 9.** Any infection in early pregnancy and risk of inflammatory bowel disease in offspring. Sensitivity analyses additionally adjusted for maternal antibiotics in pregnancy and full breastfeeding duration

|  | Main analysis  aHR (95%CI)^a^ | n | n events | aHR (95%CI)^b^ | aHR (95%CI)^c^ |
| --- | --- | --- | --- | --- | --- |
| Any infection in early pregnancy | |  |  |  |  |
| ABIS |  |  |  |  |  |
| No | — | 3,960 | 21 | — | — |
| Yes | 1.34 (0.76, 2.37) | 3,915 | 28 | 1.29 (0.72, 2.28) | 1.76 (0.85, 3.67) |
| MoBa |  |  |  |  |  |
| No | — | 51,766 | 160 | — | — |
| Yes | 1.24 (1.00, 1.55) | 49,504 | 178 | 1.23 (0.98, 1.54) | 1.24 (0.98, 1.57) |
| Pooled analyses |  |  |  |  |  |
| No | — | 55,726 | 181 | — | — |
| Yes | 1.26 (1.02, 1.55) | 53,419 | 206 | 1.24 (1.00-1.52) | 1.28 (1.03, 1.61) |

aHR: adjusted hazard ratio; CI, confidence interval. For a description of covariates, see Supplementary Table 2.

^a^Adjusted for the child’s sex, parental IBD, parental origin, maternal smoking, and maternal education level (Figure 2).

^b^Adjusted for the child’s sex, parental IBD, parental origin, maternal smoking, maternal education level, and maternal antibiotics in pregnancy.

^c^Adjusted for the child’s sex, parental IBD, parental origin, maternal smoking, maternal education level, and full breastfeeding duration.

## **Supplementary Table 10.** Any infection in early pregnancy and gastrointestinal infection in late pregnancy and risk of Crohn’s disease in offspring. Sensitivity analyses additionally adjusted for maternal antibiotics in pregnancy, and full breastfeeding duration

|  | Main analysis  aHR (95%CI)^a^ | n | n events | aHR (95%CI)^b^ | aHR (95%CI)^c^ |
| --- | --- | --- | --- | --- | --- |
| Any infection in early pregnancy | |  |  |  |  |
| ABIS |  |  |  |  |  |
| No | — | 3,960 | 6 | — | — |
| Yes | 1.84 (0.69, 4.92) | 3,915 | 11 | 1.79 (0.66, 4.85) | 3.07 (0.87, 10.8) |
| MoBa |  |  |  |  |  |
| No | — | 51,766 | 64 | — | — |
| Yes | 1.35 (0.96, 1.90) | 49,504 | 78 | 1.36 (0.97, 1.92) | 1.32 (0.93, 1.88) |
| Pooled analyses |  |  |  |  |  |
| No | — | 55,726 | 70 | — | — |
| Yes | 1.40 (1.01, 1.93) | 53,419 | 89 | 1.40 (1.01-1.94) | 1.60 (0.80, 3.22) |
| Gastrointestinal infection in late pregnancy | | |  |  |  |
| ABIS |  |  |  |  |  |
| No | — | 1,889 | 3 | — | — |
| Yes | 0.98 (0.24, 3.98) | 3,204 | 5 | 0.98 (0.25, 3.80) | 0.46 (0.14, 1.52) |
| MoBa |  |  |  |  |  |
| No | — | 87,630 | 109 | — | — |
| Yes | 2.06 (1.39, 3.04) | 13,640 | 33 | 2.06 (1.40, 3.06) | 1.99 (1.32, 2.99) |
| Pooled analyses |  |  |  |  |  |
| No | — | 89,519 | 112 | — | — |
| Yes | 1.95 (1.34, 2.84) | 16,844 | 38 | 1.91 (1.21, 3.00) | 1.07 (0.26, 4.41) |

aHR: adjusted hazard ratio; CI, confidence interval. For a description of covariates, see Supplementary Table 2.

^a^Adjusted for the child’s sex, parental IBD, parental origin, maternal smoking, and maternal education level (Figure 3).

^b^Adjusted for the child’s sex, parental IBD, parental origin, maternal smoking, maternal education level, and maternal antibiotics in pregnancy.

^c^Adjusted for the child’s sex, parental IBD, parental origin, maternal smoking, maternal education level, and full breastfeeding duration.

## **Supplementary Table 11.** The effect of maternal infections on the offspring’s risk of inflammatory bowel disease and Crohn’s disease mediated through the child’s infections and treatment with antibiotics by 12 months of age

|  |  |  | Point estimate (95% CI) | | |
| --- | --- | --- | --- | --- | --- |
|  | Exposure → mediator | Effect | ABIS | MoBa | Pooled |
| IBD | Any infection → child’s infection |  |  |  |  |
|  |  | Controlled direct effect, M=0 | 1.04 (0.00, Inf) | 1.20 (0.94, 1.53) | 1.20 (0.94, 1.53) |
|  |  | Controlled direct effect, M=1 | 1.81 (0.89, 3.68) | 0.77 (0.29, 2.08) | 1.27 (0.56, 2.90) |
|  |  | Natural direct effect | 1.81 (0.89, 3.68) | 1.24 (0.99, 1.57) | 1.29 (1.03, 1.60) |
|  |  | Natural indirect effect | 1.00 (0.95, 1.06) | 1.00 (0.98, 1.01) | 1.00 (0.99, 1.01) |
|  |  | Total effect | 1.81 (0.89, 3.70) | 1.24 (0.98, 1.57) | 1.29 (1.03, 1.61) |
|  |  | Proportion mediated | 0.01 (-0.04, 0.06) | -0.00 (-0.18, 0.15) | 0.01 (-0.04, 0.06) |
|  |  |  |  |  |  |
| IBD | Any infection → child’s antibiotic |  |  |  |  |
|  |  | Controlled direct effect, M=0 | 1.82 (0.77, 4.31) | 1.39 (0.76, 2.57) | 1.52 (0.92, 2.51) |
|  |  | Controlled direct effect, M=1 | 1.14 (0.35, 3.74) | 1.59 (0.46, 5.53) | 1.34 (0.56, 3.16) |
|  |  | Natural direct effect | 1.55 (0.78, 3.11) | 1.24 (0.99, 1.57) | 1.27 (1.02, 1.58) |
|  |  | Natural indirect effect | 1.00 (0.99, 1.02) | 1.01 (1.00, 1.02) | 1.01 (1.00, 1.02) |
|  |  | Total effect | 1.56 (0.78, 3.12) | 1.25 (0.99, 1.58) | 1.28 (1.03, 1.59) |
|  |  | Proportion mediated | 0.01 (-0.05, 0.06) | 0.04 (-0.03, 0.24) | 0.01 (-0.04, 0.06) |
|  |  |  |  |  |  |
| CD | Any infection → child’s infection |  |  |  |  |
|  |  | Controlled direct effect, M=0 | 0.87 (0.00, Inf) | 1.30 (0.91, 1.86) | 1.30 (0.91, 1.86) |
|  |  | Controlled direct effect, M=1 | 4.56 (0.98, 21.2) | 0.60 (0.09, 3.76) | 1.76 (0.24, 12.90) |
|  |  | Natural direct effect | 4.56 (0.98, 21.2) | 1.35 (0.95, 1.92) | 1.95 (0.65, 5.85) |
|  |  | Natural indirect effect | 1.00 (0.95, 1.06) | 1.01 (0.99, 1.03) | 1.01 (0.99, 1.03) |
|  |  | Total effect | 4.58 (0.99, 21.3) | 1.36 (0.95, 1.93) | 1.96 (0.66, 5.86) |
|  |  | Proportion mediated | 0.01 (0.00, 0.17) | 0.23 (-0.15, 0.24) | 0.10 (-0.12, 0.30) |
|  |  |  |  |  |  |
| CD | Any infection → child’s antibiotic |  |  |  |  |
|  |  | Controlled direct effect, M=0 | 2.03 (0.54, 7.67) | 1.35 (0.55, 3.31) | 1.53 (0.73, 3.22) |
|  |  | Controlled direct effect, M=1 | n/a | 1.36 (0.22, 8.47) | n/a |
|  |  | Natural direct effect | 2.65 (0.64, 10.1) | 1.35 (0.95, 1.92) | 1.40 (1.00, 1.98) |
|  |  | Natural indirect effect | 1.00 (0.98, 1.03 | 1.01 (0.99, 1.02) | 1.01 (1.00, 1.02) |
|  |  | Total effect | 2.66 (0.65, 10.1) | 1.36 (0.96, 1.94) | 1.41 (1.00, 1.99) |
|  |  | Proportion mediated | 0.01 (-0.02, 0.04) | 0.03 (-0.05, 0.24) | 0.01 (-0.02, 0.04) |
|  |  |  |  |  |  |
| CD | Gastrointestinal infection → child’s infection |  |  |  |  |
|  |  | Controlled direct effect, M=0 | 0.81 (0.14, 4.84) | 2.00 (1.32, 3.02) | 1.91 (1.28, 2.86) |
|  |  | Controlled direct effect, M=1 | 0.40 (0.03, 5.07) | 2.44 (0.24, 24.3) | 1.07 (0.19, 6.23) |
|  |  | Natural direct effect | 0.40 (0.03, 5.07) | 1.98 (1.32, 2.97) | 1.49 (0.45, 4.93) |
|  |  | Natural indirect effect | 1.00 (0.96, 1.04) | 1.01 (0.99, 1.04) | 1.01 (0.99, 1.03) |
|  |  | Total effect | 0.40 (0.03, 5.06) | 2.00 (1.34, 3.01) | 1.49 (0.44, 5.02) |
|  |  | Proportion mediated | 0.00 (-0.02, 0.01) | 0.03 (-0.03, 0.09) | 0.00 (-0.01, 0.02) |
|  |  |  |  |  |  |
| CD | Gastrointestinal infection → child’s antibiotic |  |  |  |  |
|  |  | Controlled direct effect, M=0 | 0.78 (0.13, 4.85) | 0.55 (0.13, 2.35) | 0.63 (0.20, 1.97) |
|  |  | Controlled direct effect, M=1 | 0.67 (0.04, 10.8) | 0.12 (0.01, 2.39) | 0.30 (0.04, 2.29) |
|  |  | Natural direct effect | 0.75 (0.16, 3.47) | 2.05 (1.36, 3.08) | 1.65 (0.73, 3.71) |
|  |  | Natural indirect effect | 0.97 (0.90, 1.05) | 0.99 (0.97, 1.00) | 0.99 (0.97, 1.00) |
|  |  | Total effect | 0.73 (0.16, 3.37) | 2.02 (1.35, 3.03) | 1.60 (0.70, 3.69) |
|  |  | Proportion mediated | 0.08 (-0.42, 0.34) | -0.03 (-0.09, 0.02) | -0.03 (-0.08, 0.03) |
|  |  |  |  |  |  |

CD, Crohn’s disease; CI, confidence interval; IBD, inflammatory bowel disease.

Controlled direct effect = The direct effect (hazard ratio) of the exposure when the mediating variable is fixed at a given value. The controlled direct effect was calculated with the mediator fixed at M=0 (not exposed) and M=1 (exposed).
Natural direct effect = The effect of the exposure (hazard ratio) when the mediating variable is fixed at its expected value in the non-exposed group.
Natural indirect effect = The effect (hazard ratio) of the non-exposed when the mediating variable changes from the expected value in the non-exposed the expected value for the exposed group.
Total effect = The total effect of the exposure, including the effect through the mediating variable (Natural direct effect * Natural indirect effect).
Proportion mediated = The proportion of the effect mediated by the mediating variable on the association between exposure and outcome.
Confidence intervals were calculated by using non-parametric bootstrap with 2,000 bootstrap replicates.

**Supplementary Table 12.** Pooled hazard ratios for childhood-onset inflammatory bowel disease (age <18 years, n=381) by maternal infections and antibiotic use in pregnancy^a^

|  | n | n events | HR | 95% CI | aHR^b^ | 95% CI |
| --- | --- | --- | --- | --- | --- | --- |
| Any infection |  |  |  |  |  |  |
| No | 39,246 | 130 | — | — | — | — |
| Yes | 77,771 | 251 | 0.93 | 0.63, 1.38 | 0.98 | 0.66, 1.45 |
| Gastrointestinal infection |  |  |  |  |  |  |
| No | 89,318 | 280 | — | — | — | — |
| Yes | 27,303 | 100 | 0.98 | 0.49, 1.95 | 0.99 | 0.47, 2.11 |
| Respiratory infection |  |  |  |  |  |  |
| No | 58,033 | 196 | — | — | — | — |
| Yes | 59,460 | 185 | 0.96 | 0.77, 1.19 | 1.00 | 0.80, 1.25 |
| Any infection in early pregnancy |  |  |  |  |  |  |
| No | 55,726 | 164 | — | — | — | — |
| Yes | 53,419 | 182 | 1.17 | 0.95, 1.45 | 1.22 | 0.98, 1.52 |
| Any infection in late pregnancy |  |  |  |  |  |  |
| No | 56,221 | 171 | — | — | — | — |
| Yes | 52,924 | 175 | 1.10 | 0.89, 1.35 | 1.13 | 0.91, 1.40 |
| Gastrointestinal infection in early pregnancy | |  |  |  |  |  |
| No | 91,950 | 284 | — | — | — | — |
| Yes | 14,413 | 52 | 1.22 | 0.90, 1.66 | 1.27 | 0.93, 1.72 |
| Gastrointestinal infection in late pregnancy | |  |  |  |  |  |
| No | 89,519 | 271 | — | — | — | — |
| Yes | 16,844 | 65 | 1.35 | 1.02, 1.78 | 1.41 | 1.06, 1.87 |
| Respiratory infection in early pregnancy | |  |  |  |  |  |
| No | 64,266 | 203 | — | — | — | — |
| Yes | 38,338 | 123 | 1.03 | 0.82, 1.28 | 1.08 | 0.86, 1.36 |
| Respiratory infection in late pregnancy |  |  |  |  |  |  |
| No | 62,665 | 203 | — | — | — | — |
| Yes | 39,939 | 123 | 0.96 | 0.77, 1.19 | 0.96 | 0.76, 1.21 |
| Any antibiotics |  |  |  |  |  |  |
| No | 89,230 | 280 | — | — | — | — |
| Yes | 26,829 | 97 | 1.13 | 0.90, 1.42 | 1.17 | 0.92, 1.48 |

aHR: adjusted hazard ratio; CI, confidence interval; HR, hazard ratio.

^a^Restricted to childhood-onset IBD (<18 years) age (n=381).

^b^Adjusted for the child’s sex, parental inflammatory bowel disease, parental origin, maternal smoking in pregnancy, and maternal education level (Supplementary Table 2).

**Supplementary Table 13.** Pooled hazard ratios for inflammatory bowel disease diagnosis excluding events of very-early-onset IBD (<6 years, n=426) by maternal infections and antibiotic use in pregnancy^a^

|  | n | n events | HR | | 95% CI | aHR^b^ | 95% CI |
| --- | --- | --- | --- | --- | --- | --- | --- |
| Any infection |  |  |  | |  |  |  |
| No | 39,246 | 155 | — | | — | — | — |
| Yes | 77,771 | 270 | 0.96 | | 0.75, 1.23 | 0.97 | 0.73, 1.29 |
| Gastrointestinal infection |  |  |  | |  |  |  |
| No | 89,318 | 316 | — | | — | — | — |
| Yes | 27,303 | 108 | 0.92 | | 0.46, 1.83 | 0.94 | 0.44, 1.99 |
| Respiratory infection |  |  |  | |  |  |  |
| No | 58,033 | 242 | | — | — | — | — |
| Yes | 59,460 | 184 | 0.94 | | 0.76, 1.16 | 0.96 | 0.77, 1.19 |
| Any infection in early pregnancy | |  |  | |  |  |  |
| No | 55,726 | 173 | — | | — | — | — |
| Yes | 53,419 | 190 | 1.15 | | 0.94, 1.41 | 1.20 | 0.97, 1.48 |
| Any infection in late pregnancy | |  |  | |  |  |  |
| No | 56,221 | 179 | — | | — | — | — |
| Yes | 52,924 | 184 | 0.99 | | 0.72, 1.36 | 0.99 | 0.69, 1.42 |
| Gastrointestinal infection in early pregnancy | | |  | |  |  |  |
| No | 91,950 | 287 | — | | — | — | — |
| Yes | 14,413 | 55 | 1.18 | | 0.87, 1.59 | 1.22 | 0.90, 1.65 |
| Gastrointestinal infection in late pregnancy | | |  | |  |  |  |
| No | 89,519 | 272 | — | | — | — | — |
| Yes | 16,844 | 70 | 1.18 | | 0.72, 1.93 | 1.21 | 0.70, 2.09 |
| Respiratory infection in early pregnancy | |  |  | |  |  |  |
| No | 64,266 | 205 | — | | — | — | — |
| Yes | 38,338 | 121 | 1.05 | | 0.75, 1.48 | 1.08 | 0.79, 1.48 |
| Respiratory infection in late pregnancy | |  |  | |  |  |  |
| No | 62,665 | 206 | — | | — | — | — |
| Yes | 39,939 | 120 | 0.91 | | 0.73, 1.14 | 0.91 | 0.72, 1.14 |
| Any antibiotics |  |  |  | |  |  |  |
| No | 89,230 | 312 | — | | — | — | — |
| Yes | 26,829 | 107 | 1.14 | | 0.91, 1.42 | 1.16 | 0.93, 1.46 |

aHR: adjusted hazard ratio; CI, confidence interval; HR, hazard ratio.

^a^Excluding cases with IBD onset before 6 years of age (n=25).

^b^Adjusted for the child’s sex, parental inflammatory bowel disease, parental origin, maternal smoking in pregnancy, and maternal education level (Supplementary Table 2).

## **Supplementary Table 14.** Pooled hazard ratios for maternal infections and antibiotic use and risk of inflammatory bowel disease in offspring additionally adjusted for delivery mode

|  | n | n events | aHR^a^ | 95% CI |
| --- | --- | --- | --- | --- |
| Any infection |  |  |  |  |
| No | 39,246 | 159 | — | — |
| Yes | 77,771 | 285 | 0.98 | 0.71, 1.36 |
| Gastrointestinal infection |  |  |  |  |
| No | 89,318 | 329 | — | — |
| Yes | 27,303 | 113 | 0.95 | 0.45, 1.98 |
| Respiratory infection |  |  |  |  |
| No | 58,033 | 245 | — | — |
| Yes | 59,460 | 199 | 1.00 | 0.81, 1.23 |
| Any antibiotics | |  |  |  |
| No | 55,726 | 326 | — | — |
| Yes | 53,419 | 112 | 1.17 | 0.94, 1.46 |

aHR: adjusted hazard ratio; CI, confidence interval. For a description of covariates, see Supplementary Table 2.

^a^Adjusted for the child’s sex, parental inflammatory bowel disease, parental origin, maternal smoking in pregnancy, maternal education level and delivery mode (Supplementary Table 2).

**SUPPLEMENTARY FIGURES**


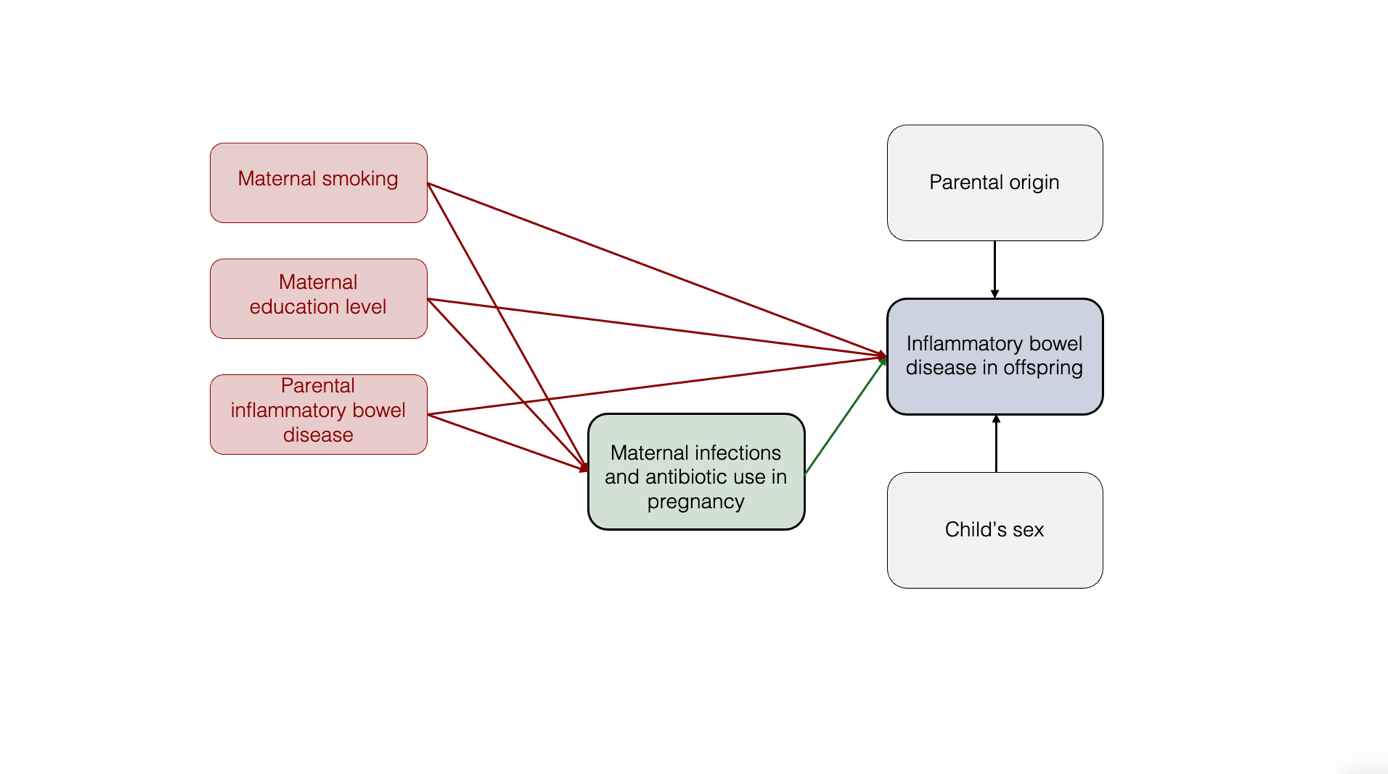


## **Supplementary Figure 1.** Directed Acyclic Graph^9^ depicting exposures (green), outcome (blue), confounding variables (red) and external factors only linked to IBD (grey) used in main analyses. Previous studies suggest an influencing effect from parental IBD,^10^ origin,^11^ and maternal education level,^12^ and smoking in pregnancy.^13^


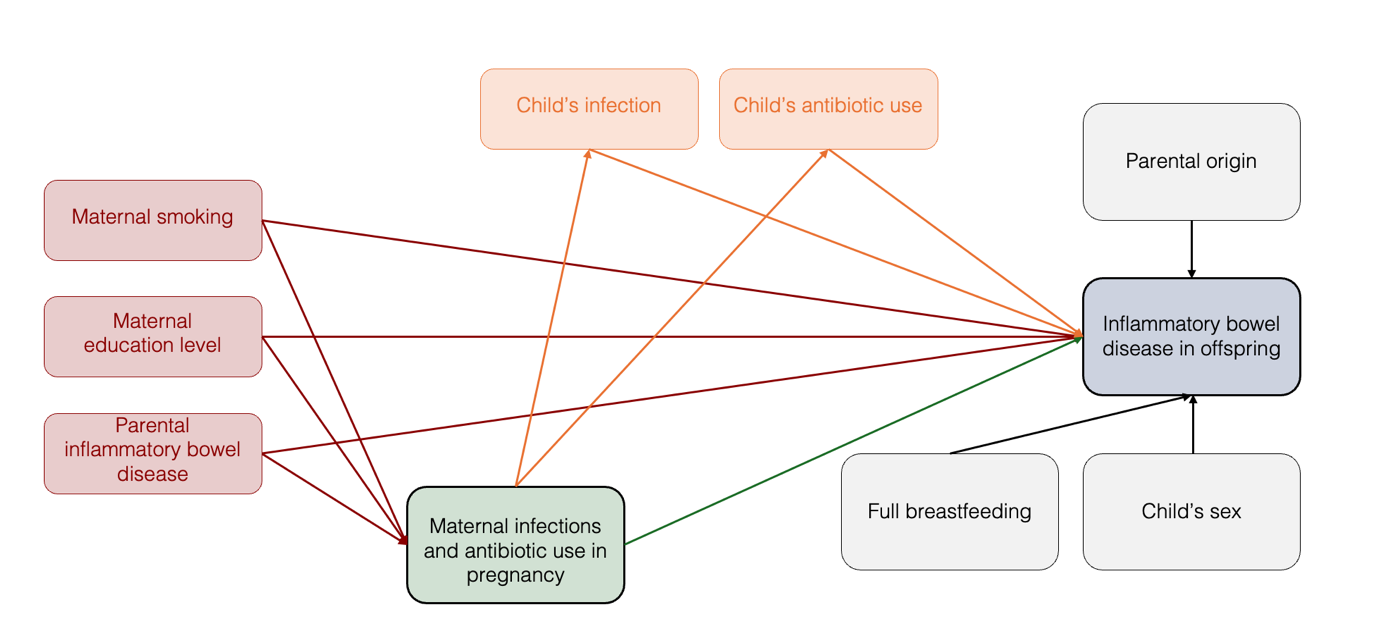


## **Supplementary Figure 2.** Directed Acyclic Graph^9^ depicting exposures (green), outcome (blue), confounding variables (red), external factors only linked to IBD (grey), and mediators (orange) used in sensitivity analyses. Previous studies suggest an influencing effect from parental IBD,^10^ origin,^11^ maternal education level,^12^ smoking in pregnancy,^13^ child’s early-life infections and antibiotic use,^14^ and breastfeeding.^15^

**REFERENCES**

1. Hutfless S, Li DK, Heyman MB, et al. Prenatal and perinatal characteristics associated with pediatric-onset inflammatory bowel disease. Dig Dis Sci 2012;57:2149-56.

2. Bernstein CN, Burchill C, Targownik LE, et al. Maternal Infections That Would Warrant Antibiotic Use Antepartum or Peripartum Are Not a Risk Factor for the Development of IBD: A Population-Based Analysis. Inflamm Bowel Dis 2017;23:635-640.

3. Örtqvist AK, Lundholm C, Halfvarson J, et al. Fetal and early life antibiotics exposure and very early onset inflammatory bowel disease: a population-based study. Gut 2019;68:218-225.

4. Torres J, Gomes C, Jensen CB, et al. Risk Factors for Developing Inflammatory Bowel Disease Within and Across Families with a Family History of IBD. J Crohns Colitis 2023;17:30-36.

5. Agrawal M, Poulsen G, Colombel JF, et al. Maternal antibiotic exposure during pregnancy and risk of IBD in offspring: a population-based cohort study. Gut 2023;72:804-805.

6. Andersen S, Hestetun SV, Bernklev T, et al. Fetal and Early Life Antibiotics and Risk of Pediatric Inflammatory Bowel Disease: A Population-based Nationwide Register Study. The Journal of Pediatrics: Clinical Practice 2024:200096.

7. Larsen JH, Andersen S, Perminow G, Stangeland HM, Mårild K, Stabell N, et al. Higher incidence of childhood-onset inflammatory bowel disease by increasing latitude in Norway, but stable incidence by age for cohorts born 2004-2012 [Accepted for Acta Paediatrica]. 2024.

8. Östensson M, Björkqvist O, Guo A, et al. Epidemiology, validation, and clinical characteristics of inflammatory bowel disease: the ABIS birth cohort study. BMC Gastroenterol 2023;23:199.

9. Suttorp MM, Siegerink B, Jager KJ, et al. Graphical presentation of confounding in directed acyclic graphs. Nephrol Dial Transplant 2015;30:1418-23.

10. Santos MPC, Gomes C, Torres J. Familial and ethnic risk in inflammatory bowel disease. Ann Gastroenterol 2018;31:14-23.

11. Misra R, Limdi J, Cooney R, et al. Ethnic differences in inflammatory bowel disease: Results from the United Kingdom inception cohort epidemiology study. World J Gastroenterol 2019;25:6145-6157.

12. Sigvardsson I, Størdal K, Östensson M, et al. Childhood Socioeconomic Characteristics and Risk of Inflammatory Bowel Disease: A Scandinavian Birth Cohort Study. Inflamm Bowel Dis 2023.

13. Sigvardsson I, Ludvigsson J, Andersson B, et al. Tobacco smoke exposure in early childhood and later risk of inflammatory bowel disease: A Scandinavian birth cohort study. J Crohns Colitis 2024.

14. Agrawal M, Sabino J, Frias-Gomes C, et al. Early life exposures and the risk of inflammatory bowel disease: Systematic review and meta-analyses. EClinicalMedicine 2021;36:100884.

15. Klement E, Cohen RV, Boxman J, et al. Breastfeeding and risk of inflammatory bowel disease: a systematic review with meta-analysis. Am J Clin Nutr 2004;80:1342-52.
